# A Systematic Screen for Tube Morphogenesis and Branching Genes in the *Drosophila* Tracheal System

**DOI:** 10.1371/journal.pgen.1002087

**Published:** 2011-07-07

**Authors:** Amin S. Ghabrial, Boaz P. Levi, Mark A. Krasnow

**Affiliations:** 1 Department of Biochemistry and Howard Hughes Medical Institute, Stanford University School of Medicine, Stanford, California, United States of America; 2 Department of Cell and Developmental Biology, University Pennsylvania School of Medicine, Philadelphia, Pennsylvania, United States of America; University of California San Francisco, United States of America

## Abstract

Many signaling proteins and transcription factors that induce and pattern organs have been identified, but relatively few of the downstream effectors that execute morphogenesis programs. Because such morphogenesis genes may function in many organs and developmental processes, mutations in them are expected to be pleiotropic and hence ignored or discarded in most standard genetic screens. Here we describe a systematic screen designed to identify all *Drosophila* third chromosome genes (∼40% of the genome) that function in development of the tracheal system, a tubular respiratory organ that provides a paradigm for branching morphogenesis. To identify potentially pleiotropic morphogenesis genes, the screen included analysis of marked clones of homozygous mutant tracheal cells in heterozygous animals, plus a secondary screen to exclude mutations in general “house-keeping” genes. From a collection including more than 5,000 lethal mutations, we identified 133 mutations representing ∼70 or more genes that subdivide the tracheal terminal branching program into six genetically separable steps, a previously established cell specification step plus five major morphogenesis and maturation steps: branching, growth, tubulogenesis, gas-filling, and maintenance. Molecular identification of 14 of the 70 genes demonstrates that they include six previously known tracheal genes, each with a novel function revealed by clonal analysis, and two well-known growth suppressors that establish an integral role for cell growth control in branching morphogenesis. The rest are new tracheal genes that function in morphogenesis and maturation, many through cytoskeletal and secretory pathways. The results suggest systematic genetic screens that include clonal analysis can elucidate the full organogenesis program and that over 200 patterning and morphogenesis genes are required to build even a relatively simple organ such as the *Drosophila* tracheal system.

## Author Summary

Elucidating the genetic programs that control formation and maintenance of body organs is a central goal of developmental biology, and understanding how these programs go awry in disease has important implications for medicine. Many such organogenesis genes have been identified, but most are early-acting “patterning genes” encoding signaling proteins and gene regulators that control expression of a poorly characterized set of downstream “morphogenesis genes,” which encode proteins that generate the remarkable organ forms and structures of the constituent cells. We screened ∼40% of the fruit fly *Drosophila* genome for mutations that affect tracheal (respiratory) system development. We included steps to bypass complexities from mutant effects on other tissues and steps to exclude mutations in general cell “housekeeping genes.” We isolated mutations in ∼70 genes that identify major steps in the organogenesis program including an integral cell growth control step. Many of the new tracheal genes are “morphogenesis genes” that encode proteins involved in cell structure or intracellular transport. The results suggest that genetic screens can elucidate a full organogenesis program and that over 200 patterning and morphogenesis genes are required to build even a relatively simple organ.

## Introduction

Elucidating the genetic programs of organ formation and maintenance is a central goal of developmental biology and medicine. Many organogenesis genes have been isolated in systematic genetic screens in model organisms, and many others have been identified by their organ-selective expression patterns and by candidate gene analysis. These approaches have been very successful at discovering the signaling pathways and transcription factors that induce and pattern organs and specify cell fates, but they have been much less successful at identifying the downstream effectors that execute morphogenesis programs, what we call morphogenesis genes [1], [2], [3], [4]. A similar abundance of signaling and transcription factor genes and dearth of morphogenesis genes has obtained from the pioneering genetic dissection of *Drosophila* body axis formation and other early developmental events [5]. We reasoned that many morphogenesis genes would function in multiple organs and developmental processes, so mutations in these genes would be pleiotropic and hence discarded in most genetic screens. We therefore designed systematic, saturation screens for genes required for *Drosophila* tracheal system organogenesis that included clonal analysis of gene function in the tracheal system, to identify all tracheal genes including those with pleiotropic phenotypes. The results of a screen of the third chromosome, representing ∼40% of the *Drosophila* genome [6], are described here, and the results of a first (X) chromosome screen initiated earlier will be described elsewhere ([7]; M. Metzstein and M.A.K., unpublished data).

The *Drosophila* tracheal (respiratory) system is a branched tubular network that transports oxygen throughout the body [8]. It is one of the most intensively studied and best understood organogenesis programs [9], [10], and it has emerged over the past decade as a paradigm of branching morphogenesis, the developmental process that gives rise to many organs including the lung, vascular system, kidney, and pancreas. Understanding how branching networks are patterned and how cellular tubes are made, shaped, and maintained is of fundamental importance in cell and developmental biology, and in medicine for understanding and treating tubular diseases such as aneurysms and polycystic kidney disease.

**Figure 1:**
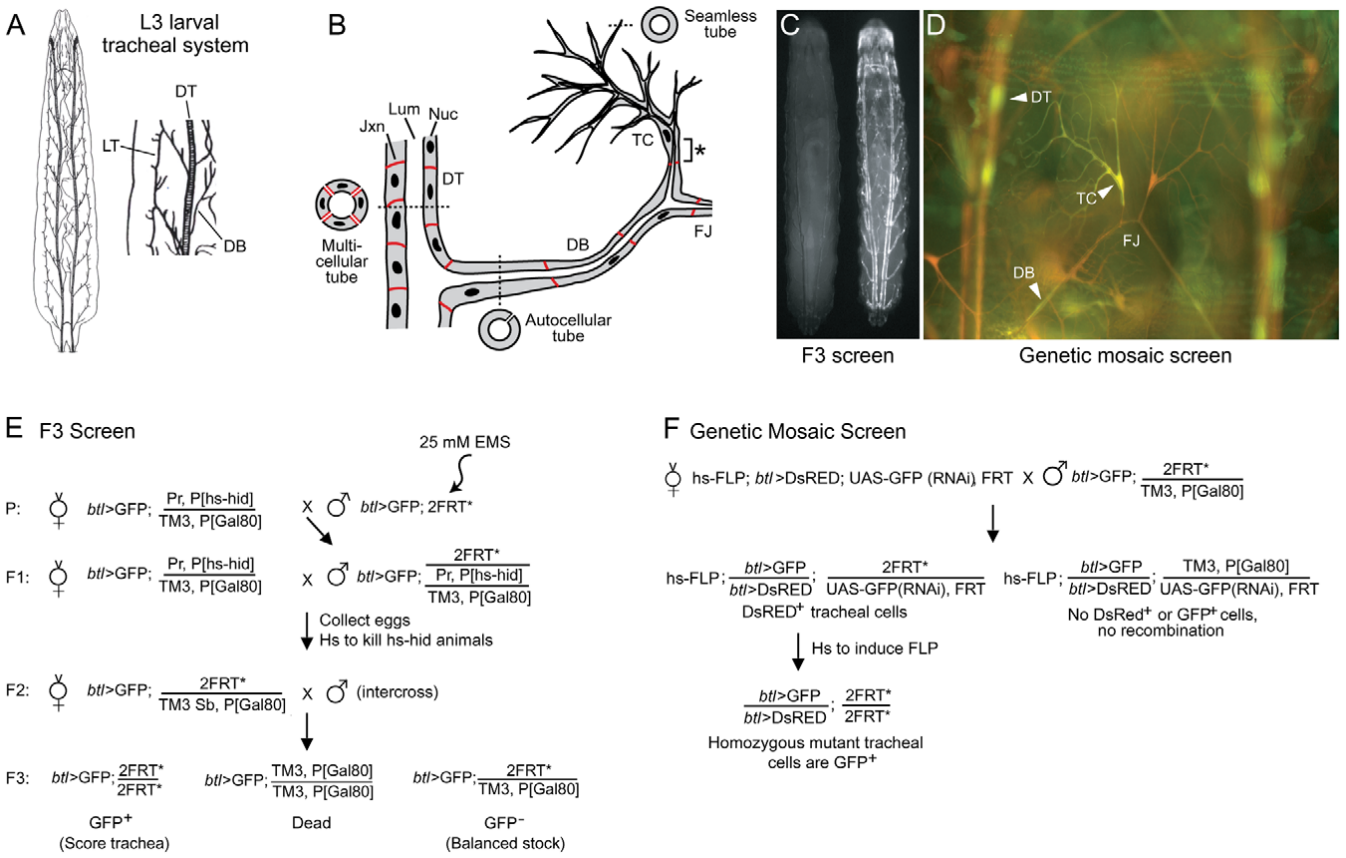
Design of tracheal mutant screen. (A) Diagram of *Drosophila* tracheal system in third instar larva (dorsal view, anterior up unless noted otherwise). A close up of two hemisegments (Tr4 and Tr5) are shown at right, with some primary branches indicated. DT, dorsal trunk; DB, dorsal branch; LT, lateral trunk. (B) Schematic showing cellular structure of dorsal trunk and dorsal branch. Dashed lines indicate plane of section of cross-sections shown. DT is a multicellular tube with multiple cells and intercellular junctions seen in cross-section. DB stalk is an autocellular tube, a single cell wrapped around the luminal space and sealed by an autocellular junction. DB terminal cell (TC) forms multiple terminal branches, each of which is a “seamless” tube lacking junctions. The base of the terminal cell (*), from its junction with a stalk cell to the nucleus, is an autocellular tube. The fusion joint (FJ) is the position where two fusion cells, each of which forms a seamless tube, connect contralateral tracheal hemisegments. Lum, tracheal lumen (black); Jxn, intercellular junctions (red); Nuc, cell nuclei (black). (C) Fluorescence micrograph of two sibling F3 larvae from the F3 screen diagrammed in panel E. The GFP^-^ larva at left is heterozygous for the mutagenized third chromosome; it is nearly invisible because it contains, in trans to the mutagenized chromosome, a Gal80-expressing balancer chromosome that prevents expression of btl-Gal4, UAS-GFP (btl>GFP). The GFP^+^ larva at right is homozygous for a mutagenized third chromosome; it lacks the Gal80 chromosome, so expresses GFP throughout the tracheal system. (D) Fluorescence micrograph of a segment (Tr5) of the tracheal system from a third instar larva generated by the genetic mosaic strategy shown in panel F. All tracheal cells express btl>DsRED (red); homozygous clones lack the UAS-GFP(RNAi), so express in addition btl>GFP (green). Dorsal trunk (DT), dorsal branch (DB) and terminal cell (TC) clones are marked (arrowheads). Dorsal branch fusion joint (FJ) connecting the left and right hemisegments is indicated. (E) Genetic scheme of F3 screen. EMS, ethyl methanesulfonate; Pr, Prickly; P[hs-hid], heat shock inducible *hid* transgene; TM3, third chromosome balancer; P[Gal80], transgene with ubiquitous tubulin promoter driving expression of Gal80, a Gal4 inhibitor; 2FRT, two Flp Recombinase Target (FRT) site transgenes (FRT2A on 3L and FRT82B on 3R) flanking the third chromosome centromere; Sb, Stubble; *, mutagenized chromosome. (F) Genetic scheme of the mosaic screen. hs-FLP, heat-inducible FLP recombinase transgene; UAS-GFP(RNAi), Gal4-inducible (Gal4 upstream activating sequence) GFP RNAi transgene.

The tracheal system develops from 10 pairs of tracheal sacs that arise by invagination of the embryonic ectoderm [8], [11]. Each sac is an epithelial monolayer composed of ∼80 cells. Primary tracheal branches are formed by groups of 3–20 cells that bud from the sacs in different directions and successively sprout secondary and terminal branches. Some specialized primary and secondary branches grow towards and fuse with branches from neighboring sacs to interconnect the tracheal network [12]. The transformation of the simple epithelial sacs into an extensively branched tubular network occurs without cell proliferation, and is mediated by cell migration, rearrangement, and dramatic changes in cell shape [11], [13], [14], [15]. During embryogenesis, the lumens of the developing tracheal branches are filled with a complex and changing matrix, which is cleared and replaced with gas just before the embryo hatches and the tubes become functional in respiration [8], [16]. In the larva, terminal cells ramify extensively to form many new terminal branches (tracheoles), long cytoplasmic extensions that grow toward oxygen-starved cells and then form a cytoplasmic, membrane-bound lumen, creating tiny tubes (<1 um diameter) that supply the targets with oxygen (Figure 1B) [14], [17]. Unlike primary (multicellular) and secondary (unicellular) branches, tubes sealed by intercellular and autocellular junctions (Figure 1B), terminal branches lack cell junctions and resemble the “seamless” endothelial tubes of the mammalian microvasculature [18], [19], [20] and *C. elegans* excretory system [21].

The first important tracheal gene identified was *trachealess*, isolated in the classical screens for embryonic patterning mutants by the complete and selective absence of the tracheal system [22] and later shown to encode a bHLH-PAS transcription factor, the earliest expressed tracheal-specific gene and a master regulator of tracheal identity [23], [24]. Ten years after the discovery of *trachealess*, a *Drosophila* homolog of mammalian FGFRs was isolated and named Breathless because it is selectively expressed in the developing tracheal system and required for branching [25], [26], [27]. Around this time the first systematic screens for tracheal mutants were conducted, screens of P[lacZ] insertions that identified about 50 tracheal genes that subdivided embryonic tracheal development into genetically distinct processes including primary, secondary, and terminal branching, branch fusion, and tube size control [11], [28]. Mapping and molecular characterization of these genes identified many components and modulators of the Breathless FGFR signaling pathway. These include Branchless FGF, which activates Breathless FGFR and plays a central role in controlling and coupling each of these processes by guiding outgrowth of primary branches and inducing expression of key genes encoding transcription factors such as *pointed*, *blistered/pruned*, and *escargot* required, respectively, for secondary and terminal branching and branch fusion [12], [14], [26], [29]. Many other important genes have been identified by their tracheal expression patterns, analysis of candidate genes, and serendipitous discovery of a tracheal function for genes initially studied in other contexts [30], [31], [32], [33]. And, over the past several years, several screens of chemically-induced mutations for tracheal morphogenesis defects in embryos and larval tracheal and air sac primordium clones have been conducted [34], [35], [36] along with more targeted genetic and genomic screens for genes that are expressed or function downstream of some of the key early signaling pathways (*branchless, breathless*) and transcription factors *(trachealess*, *ribbon)* [37], [38], [39], [40]. Together these approaches have implicated ∼100 genes in tracheal development, most of which encode transcription factors or components of signaling pathways (FGF, TGFα/EGF, TGFβ, Wnt, Notch, Slit/Robo, Jak/Stat, and Hedgehog) [1], [2], [4], [9] (Table S1). However, the downstream targets of the signaling pathways and transcription factors, the morphogenesis and maturation genes that create, shape, and stabilize the tubes, have only recently begun to be identified. And, although expected to be a large class, they are substantially under-represented among characterized tracheal genes (Table S1) [4].

We conducted a large-scale screen of chemically-induced mutations to assess the function of nearly all *Drosophila* third chromosome genes, including early essential genes and genes with pleiotropic phenotypes. We sought to identify most or all of the genetically separable steps in tracheal development; to identify new tracheal genes associated with each step; and to provide an estimate of the total number of genes required to build an organ. We were especially interested in identifying tracheal morphogenesis genes. Our approach involved clonal analysis in the tracheal system of all chemically-induced mutations that did not survive late enough in development as homozygotes to assess their tracheal function, and a secondary screen to exclude general “house-keeping” genes. We isolated mutations representing ∼70 genes, 14 of which we identified molecularly, implicating most of the genes as morphogenesis genes and revealing new cell biological pathways in tracheal development. Many of the mutations affect terminal branch morphogenesis, genetically subdividing this poorly understood process into five major morphogenetic steps including an integral cell growth step.

## Results

### Screen design

To identify tracheal morphogenesis genes, we screened the third chromosome for EMS-induced mutations that affect larval tracheal morphology. Approximately 4,300 mutagenized third chromosomes were generated, and balanced lines were established for each. Three-quarters (73%) of the lines were homozygous lethal. Assuming a Poisson distribution, there were ∼1.3 lethal mutations per mutagenized third chromosome and a total of 5600 lethal mutations screened. Because there are ∼3600 essential *Drosophila* genes [6], with roughly 40% (∼1370) on the third chromosome [41], we expected to obtain an average of about four (5600/1370) mutations per gene, with at least one mutation in 97% of all third chromosome genes.

The mutants were screened for tracheal defects in two steps. First, homozygous third instar larvae of the F3 generation (Figure 1E), which carried *btl*-GAL4 and UAS-GFP transgenes (abbreviated *btl*>GFP) to label tracheal cells, were scored for tracheal defects by fluorescence microscopy. The balancer chromosome carried a Tub-GAL80 transgene that inhibits Gal4 and blocks expression of UAS-GFP so only homozygous mutant animals expressed GFP, facilitating screening (Figure 1C). For 40% of lines, GFP^+^ F3 third instar larvae were not recovered, presumably because the homozygous mutations caused early lethality.

These pre-pupal lethal lines were analyzed in a second step of the screen, using a genetic mosaic strategy in which we examined clones of homozygous mutant tracheal cells in otherwise heterozygous larvae (Figure 1F). We devised a variant of the MARCM clone marking strategy [42] employing a UAS-GFP(RNAi) transgene on the homologous chromosome, *in trans* to the mutation of interest, which allowed us to label all tracheal cells with *btl*>DsRed and homozygous mutant tracheal cells (lacking UAS-GFP(RNAi)) with *btl*>GFP (Figure 1D). This facilitated comparison of homozygous mutant cells (DsRed^+^, GFP^+^) with surrounding wild type tracheal cells (DsRed^+^, GFP^-^), enhancing the sensitivity of the screen and detection of cell non-autonomous effects in the tracheal system.

### Overview of screen results

Over 600 mutants with highly penetrant and expressive tracheal defects were identified. However, the vast majority were lethal mutations in genes required cell autonomously for tracheal cell growth and survival, and later found in a secondary screen (see below) to be presumptive housekeeping genes and discarded. 133 tracheal mutations were saved (Table 1 and Table S3), 18 from the F3 screen (alleles with two letter prefix, e.g. PC213) and 115 from the genetic mosaic screen (no prefix).

**Table 1:** Tracheal morphogenesis mutant collection.

Name (alleles^1^)	Tracheal Phenotype (category) [other affects]	Map Position (method^2^)
*appaloosa* (771)	TC gas-filling defect, variable penetrance (5A1). [Abundance of epidermal clones; ectopic bristles in trans to Df35]	87F9; 87F12 (Df 1,2)
*asthmatic* (742, 1530)	TC gas-filling defect (5A1). Incompletely penetrant autocellular gas-filling defect (5B).	3R (MA)
*balloon* (1448)	DT cell clones with dilated lumen (4C1). Variable TC shape defect, mild pruning, rare multi-lumen defect.	70C; D (Df 11,12)
*black hole* (538)	Large cytoplasmic vacuoles in TC (4A5). Variable TC pruning and sporadic odd positioning of nucleus.	64C; 65C (Df 3)
*braided* (1615)	Variable TC multi-convoluted lumen defect (4A3). Moderate pruning.	3L (MA)
*bulgy* (636)	Dorsal trunk clones with lumen dilation (4C1). TC pruned with gas-filling defect.	83C;D (Df 4)
*burs* (942, 1139)	Selective TC pruning (3C1). 942 allele with TC gas-filling defect.	73D1 (Df 5)
*carbuncle* (804)	Large GFP-excluding bodies in all mutant tracheal cells (4A5). TC lumen formation is variably discontinuous (4A1).	66B; 66C (Df 6,7)
*cincher* (773)	Dorsal trunk cell clones are tiny (2C)	83B7; 83C2 (Df 8)
*conjoined* (356)	Thick and severely pruned TC (3C2). Defective intercalation/autocellular tube formation (4B). Auto/subcellular tube gas-filling defect (5A3). DT cells rounded and contribute minimally to multicellular lumen.	3R (MA)
*constricted* (960)	Dorsal trunk lumen constriction (4C2)	70C; D (Df 11,12)
*creeper* (153)	Variable TC multi-convoluted lumen defect (3A). Mild to moderate TC pruning (3A).	96A (Df 9, 10)
*corset* (897)	Dorsal trunk cell clones are tiny (2C)	3L-4 lethals (Df 14-18)
curlicue (1629)	TC tips with variable multilumen defect (4A3). TCs moderately pruned.	3L (MA)
*cystic lumens* (1243)	Dramatic TC lumen dilation defect (4A4). Moderate TC pruning.	63F6; 64C15 (Df 19)
*dark matter* (1417)	Cytoplasmic vacuoles (4A5). Moderate TC pruning and lumens slightly convoluted.	84B;D (Df 20)
*denuded* (PC213, 1520)	Selective TC pruning (3C1). Lumens in remaining branches have small bore.	*cu-sr* (RM)
*Disjointed* (169)	TC autocellular-seamless tube junction gas-filling defect (5A3). Mild to moderate pruning.	3R (MA)
*dyspneic* (1348, 1359)	Strong TC gas-filling defect (5A1) and autocellular tube gas-filling defect (5B). DT cells show mild lumen constriction (4C2).	3R (MA)
*etiolated* (1537, 1736)	Tracheal-specific growth defect (2B). [Normal size eyes in EGUF/hid assay]	73A; 74F (Df 21)
*failed fusions* (PA14, AZ63)	Dorsal branch and sporadic lateral trunk fusion defects (1C)	3R (MA)
*flash flood* (AP67)	Lateral trunk fusion and clearance defect in mutant larvae (5B)	Ch 3
*ichorous* (206)	TC gas-filling defect with lumGFP accumulation (5A1)	85A2; 85C1-2 (Df 22 )
*impatent* (1472, 1490, 1757)	TC lumen formation defective with lumGFP accumulating in puncta (4A1)	61 (Df 23)
*ivy* (1781)	TC moderately to severely pruned with multiple convoluted lumens (4A3)	65A,B; 66B,C & 70E;71F (Df 6,7,15,16, 24)
*jolly green giant* (1149)	All mutant tracheal cells overgrown with most dramatic effect on TCs (2A). [Distal hairy-wing in trans to Df9]	95D; 95F & 98E; 99A (Df 25,26)
*liquid-filled* (725)	TC defective for gas-filling (5A1)	82F (Df 27)^3^
*littoral* (762)	Variable TC gas-filling defect, with tips most often affected (5A2)	3R (MA)
*loose caboose* (AB56)	DB10 fusion defect with posterior spiracles often misaligned (1C)	Ch 3
*lopped* (784)	Moderate to strong TC pruning (3A)	82F (Df 27)
*lotus* (312)	TC pruned and sometimes appears fragmented (as if degenerating) and other tracheal cells are small (2B). TC seamless/autocellular tube connection is defective (5A3). TC rounded and contributes minimally to lumen. [Normal size eyes in EGUF/hid assay]	3R (MA)
*miracle-gro* (338, 878, 1483, 1489)	All mutant cells overgrown with most dramatic effect on TC (2A)	99F; 100B (Df 28)
*missing parts* (AG33)	Region-specific TC loss and fusion defect (1B).	Ch 3
*moon cheese* (1524)	All cells accumulate GFP-excluding vacuoles (4A5). TC pruned with variable lumenal discontinuities (4A1).	64C;D (Df 29)
*no tc clones-L* (602, 724, 788, 1118, 1187, 1476, 1684, BN40)	Mutant cells never occupy TC position (1A)	*h-th (*RM)
*no tc clones-R* (198, 1318)	Mutant cells never occupy TC position (1A). When clones present near branch tip, TC often missing.	94D; 95A (Df 30)
*oak gall* (696)	Thick and severely pruned TC (3C2). Defective intercalation/autocellular tube formation (4B). Auto/subcellular tube gas-filling defect (5A3). DT cells rounded and contribute minimally to multicellular lumen.	3R (MA)
*paltry* (1181, 1803)	Severe TC pruning (3A), sometimes appear to be degenerating. Variable gas-filling defect.	63F; 64C & 68A; 69A (Df 19, 31)
*panting* (1318, 1584)	Gas-filling defect at TC branch tips (5A2). Possible mild TC pruning.	77B-C; 77F-78A (Df 32)
*piddling* (1002, 1834)	Mild to moderate TC pruning (3A) with variable gas-filling defect	69C; F (Df 33)
*scrub* (659)	Severe TC pruning (3A). Gas-filling defect with no visible lumGFP (5).	64C; 65C & 76B; 77B (Df 42,51)
*short of breath* (360, 404, 483, 791, 1705)	Strong TC gas-filling defect (5A1). Autocellular tubes also show gas-filling defect (5B). DT cells show mild lumen constriction (4C2).	82F (Df 27)
*short round* (1103, 1695)	Moderate to severe TC pruning with incomplete gas filling (3A). DT cells are small and rounded.	87B; 87D (Df 34,35)
*small potatoes* (1113, 1166, 1694)	Moderate TC pruning (3A) and incomplete gas filling. DT lumen bulges outward (4C1).	95A; D, 96A; B, & 97A; 98A (Df 36,37,38)
*spikes* (735)	Mild TC pruning defect but branches show excess filopodia (3C)	3R (MA)
*sprout* (574)	All tracheal cells are tiny, but TC are nevertheless robustly branched (2B)	3R (MA)
*steeple* (A187)	Dorsal branch TC missing with high penetrance in homozygous larvae (1B)	Ch 3
*stertorous* (1290, 1321)	All seamless tubes defective for gas-filling (5A1)	65F3; 66B10 (Df 16)
*tendrils* (666, 1308, 1469, 1539)	TC pruning with multiple convoluted lumens (4A3)	*ru-h* (RM)
*tiny tubes* (630, 1309)	Mild to moderate TC pruning with narrow bore lumens (3A)	83A6; 83B6 (Df 39,40)
*topiary* (700, 1019)	Moderate (1019) to severe (700) TC pruning (3A)	61A; D3 & 64C; 65C (Df 41,42)
*truncated* (533, 1659)	Moderate TC pruning (3A) & incomplete gas-filling	69A2-3 (Df 31,43)
*vine* (512)	TC with multiple convoluted lumens (4A3) and moderate pruning	89E (Df 44,45)
*wavy lumens* (894)	TC with tortuous lumens (4A2) and moderate pruning	3R (MA)
*whacked* (PC24, 220)	Variable TC pruning and lumen formation defect, including prematurely truncated tubes ending in local dilations and discontinuous tubes (4A1, 4A4).	86 E14; 86E17 (Df 47)
*wheezy* (770)	Mild TC pruning (3A) and air filling defect. DT clones show darker cuticle over apical membrane. [Cross-veinless wing defect *in trans* to Df 13]	89E11; 90A7 (Df 46,49)
*winded* (613, 1227, 1375, 1508)	Strong TC pruning (3A) and air filling or seamless tube formation defect	65F3; 66B10 (Df 16)
*wobbly lumens* (BG13)	Tortuous lumens in TC branches of third instar larvae (4A2)	Ch 3

1 Allele names beginning with two letters (e.g. PC213) were identified in the F3 screen. All others were identified in the genetic mosaic analysis.

2 Methods used for mapping: MA, mosaic analysis; RM, meiotic recombination mapping with recessive markers; Df, deficiency mapping with failure to complement deficiencies indicated; Ch 3, unmapped mutation on the third chromosome. Chromosomal deficiencies used: (1) Df(3R)126c, (2) Df(3R)Urd, (3) Df(3L)ZN47, (4) Df(3R)EXEL7284, (5) Df(3L)EXEL9002, (6) Df(3L)ZP1, (7) Df(3L)66C-G28, (8) Df(3R)EXEL7283, (9) Df(3R)crb87-5, (10) Df(3R)XS, (11) Df(3L)fz-GF3b, (12) Df(3L)fz-CAL5, (13) Df(3R)MAP11, (14) Df(3L)ru-22, (15) Df(3L)RM5-2, (16) Df(3L)pbl-X1, (17) Df(3L)AC1, (18) Df(3L)ED230, (19) Df(3L)GN24, (20) Df(3R)Antp17, (21) Df(3L)81k19, (22) Df(3R)p-XT103, (23) Df(3L)bab-PG, (24) Df(3L)Brd6, (25) Df(3R)crb-F89-4, (26) Df(3R)3450, (27) Df(3R)3-4, (28) Df(3R)tll-g, (29) Df(3L)EXEL6105, (30) Df(3R)M95A, (31) Df(3L)vin5, (32) Df(3l)ri-79c, (33) Df(3L)ED4486, (34) Df(3R)KarD2, (35) Df(3R)ry615, (36) Df(3R)mbc-R1, (37) Df(3R)96B, (38) Df(3R)Tl-P, (39) Df(3R)Dr-rvl, (40) Df(3R)01215, (41) Df(3L)emc-E12, (42) Df(3L)ZN47, (43) Df(3L)F10, (44) Df(3R)Spf, (45) Df(3R)EXEL6270, (46) Df(3R)C4, (47) Df(3R)EXEL6276, (48) Df(3R)tll-e, (49) Df(3R)ED5780, (50) Df(3R)EXEL6274, (51) Df(3L)XS533, (52) Df(3L)GN34, (53) Df(3R)Win11, (54) Df(3R)ry27, (55) Df(3R)XF3, (56) Df(3L)AC1, (57) Df(3R)DG4 , (58) Df(3R)Cha7.

3 Fails to complement l(3)82Fa.

Other abbreviations: TC, terminal cell; DT, dorsal trunk.

Most of the mutations could be placed into one of five phenotypic classes (Table 1 and Table S3): (1) cell selection/specification; (2) cell size; (3) branch number, pattern size and shape; (4) tube formation, number, position, and shape; and (5) lumen clearance/gas filling. Within each broad class, phenotypic subgroups were defined and representative mutations in each subgroup were selected and subjected to detailed phenotypic characterization and genetic mapping as detailed below. Some mutations had more than one defect and were placed into more than one subgroup or class. Only a few mutants with cell non-autonomous effects were recovered from the clonal screen (see below), implying that such tracheal mutations are rare.

### Genetic complementation groups

Complementation tests allowed assignment of 68 mutations to 24 loci (Table 1). In addition, four mutations that were mapped to specific chromosomal deficiencies were found to be new alleles of extant genes in the mapped intervals (see below). In addition to these 72 definitively assigned mutations in 28 loci, we also characterized and named 30 other mutations with interesting tracheal phenotypes (Table 1). The rest of the saved mutations (Table S3) were not extensively characterized; 12 of these are associated with mapped lethal mutations that complement extant tracheal mutations in the mapped interval, so may represent additional essential tracheal genes.

It is difficult to estimate the number of mutations we obtained in previously known tracheal genes because the mosaic loss of function phenotype is not known for most tracheal genes, and the number of complementation tests necessary to determine this number directly is prohibitive. However, the apparent absence of mutations in two known tracheal genes (*stumps* and *trachealess*) whose mosaic phenotype we determined, and the lower than expected allele frequencies (mean 2.6) obtained for the 28 definitively identified loci, indicate that the screen did not achieve the degree of saturation predicted by a Poisson distribution. Nevertheless, the screen was extensive so we think it is likely it identified mutations in most processes and molecular pathways involved in tracheal tube morphogenesis.

Below, we describe each of the major phenotypic classes and subcategories, and representative mutations in each. Most of the mutations are homozygous lethal and all caused highly penetrant and expressive tracheal phenotypes. For ease in presentation, we treat the strongest phenotype in each complementation group as the null phenotype; however, we do not know for most if they truly represent the null condition because it is not readily possible to generate hemizygous (mutant/deficiency) clones for comparison or to exclude partial masking of phenotypes due to perdurance of wild type protein in mutant cells.

### Class 1: cell selection/specification mutants

These mutations eliminated specific tracheal cell types or blocked their differentiation.

#### No mutant terminal cells (1A)

**Figure 2:**
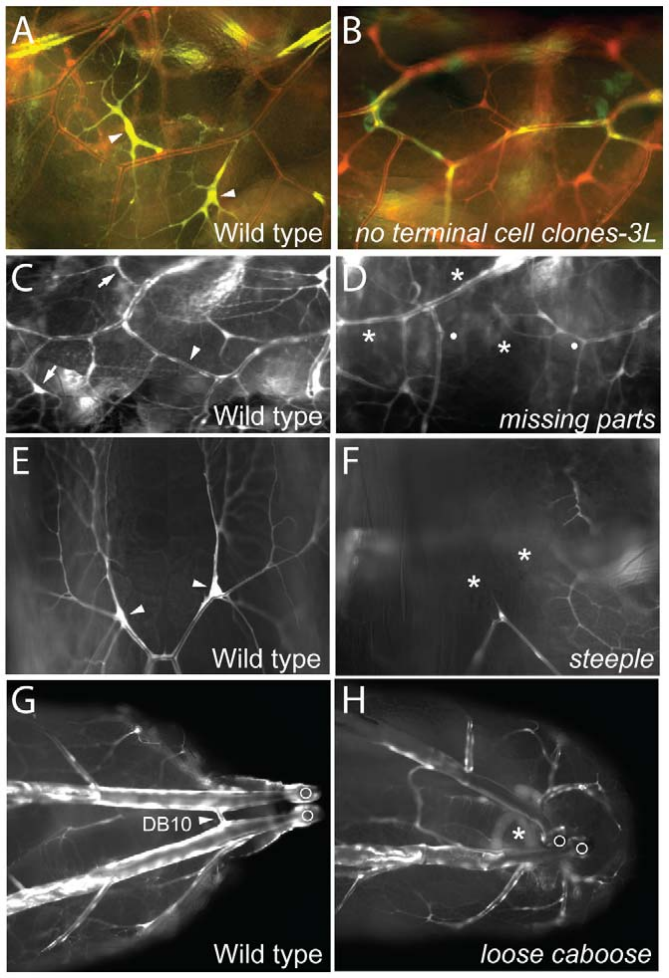
Tracheal cell selection/specification mutants. (A, B) Lateral views (anterior left) of a portion of the lateral tracheal trunk (between two transverse connectives) of genetic mosaic third instar larvae with control wild-type clones (A) and homozygous *no terminal cell clones-3L* clones (B). All tracheal cells express DsRed (red) and tracheal clones also express GFP (green) so appear yellow. Terminal cell clones (arrowheads) are present in A but absent in B. (C–H) Portions of the tracheal system of wild type control and mutant third instar larvae homozygous for the mutations indicated. (C, D) Lateral views (anterior left) of wild type (C) and *missing parts* mutant (D). Tracheae are labeled with GFP (white). Positions of two normal terminal cells (arrows) and a lateral trunk (LT) fusion joint (arrowhead) are indicated in C. In D, the corresponding terminal cells and LT fusion joint are missing (*), with broken ends of LT indicated by white dots. (E, F) Dorsal views of distal ends of a pair of dorsal branches labeled with GFP (white) in wild type (E) and *steeple* mutant (F). Note terminal cells (arrowheads in E) are missing (*) in *steeple* mutant (F). (G, H) Dorsal view of posterior of wild-type (G) and *loose caboose* mutant (H) with tracheae labeled with GFP (white). Arrowhead, position where contralateral dorsal branches (Tr10) connect to form the DB10 fusion joint (G). DB10 fusion joint is missing (*) in H; in the absence of the fusion joint, the positions of the disconnected parts of the tracheal system are more variable. Open circles, posterior spiracles.

Two complementation groups (*no terminal cell clones-3L* and *no terminal cell clones-3R*) gave normal numbers of mutant clones in the mosaic screen but the mutant cells rarely if ever included terminal cells (Figure 2A, 2B). This novel phenotype lead to new insights into the terminal cell selection process (see Discussion).

#### Region-specific terminal cell loss (1B)

Two lines lacked terminal cells in specific body regions of homozygous mutant animals. In *missing parts* (AG33) mutants, dorsal branch, fat body, and CNS terminal cells were frequently missing, but terminal cells in other positions were unaffected (Figure 2C, 2D). *steeple* (AI87) mutants frequently lacked dorsal branch terminal cells (Figure 2E, 2F), although all other terminal cells were present. These phenotypes demonstrate that there are region- or branch-specific modulators of terminal cell selection, specification, or survival, the existence of which had been suggested by marker expression patterns [11].

#### Failed branch fusions (1C)

Four lines showed frequent branch fusion failures in homozygous larvae. These included *failed fusions* (PA14, AZ63) and *missing parts* (Figure 2D), which showed dorsal branch and lateral trunk fusion defects as well as the terminal cell defects noted above. *loose caboose* (AB56) mutants had frequent defects in fusion of the most posterior dorsal branches (Figure 2G, 2H).

### Class 2: cell size mutants

These mutants had their most profound affects on tracheal cell size. For nearly all mutations, the effect on terminal cell size correlated with branch number: larger cells had more terminal branches and smaller cells had fewer. One exceptional mutant, *sprout,* is presented below.

#### General tracheal cell overgrowth (2A)

**Figure 3:**
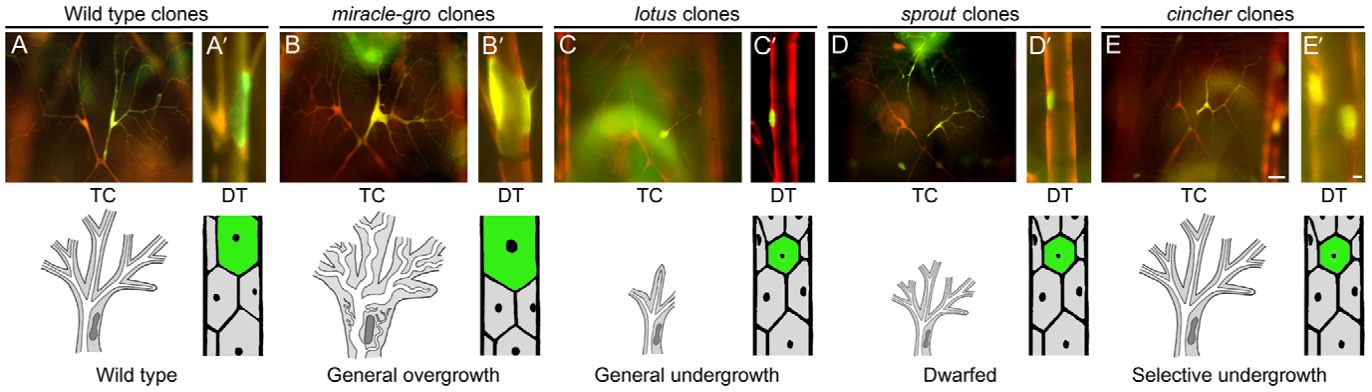
Tracheal cell size mutants. Micrographs (top panels) and schematics (lower panels) of genetic mosaic third instar larva showing terminal cell (TC, A–E) and dorsal trunk (DT, A'–E') clones (GFP^+^, green; at right) of control wild type (A, A'), *miracle-gro^338^* (B, B'), *lotus^312^* (C, C'), *sprout^574^* (D, D'), and *cincher^773^* (E, E') cells. In A–E, a contralateral control heterozygous terminal cell (DsRED^+^, red; at left) is included for comparison. The maximal soma cross-sectional area of *miracle-gro^338^* terminal cell clones (0.87±0.05 units in Image J (mean±SEM), n = 10 clones) was four-fold greater than that of wild type control terminal cell clones (0.22±0.03 units). Extra branches in the *miraclo-gro* clone are highlighted in Figure 4C/4C'. Bar, 50 µm (A–E), 10 µm (A'–E').

Two complementation groups showed tracheal cell overgrowth phenotypes in the mosaic screen, and the enlarged terminal cells had more branches (see below). *miracle-gro* (338,878,1483,1489) mutant cells were several times larger than normal (Figure 3A, 3B), and the lumens of the mutant cells had larger bores and pursued a more tortuous path through the cytoplasm. The latter phenotype was fully penetrant in the seamless tubes of terminal cells and partially penetrant in larger tracheal tubes. *jolly green giant* (1149) caused a more subtle increase in cell size, most readily detected in terminal cells, and no obvious alteration in lumen morphology. Both *miracle-gro* and *jolly green giant* mutations also cause overgrowth of cells outside the tracheal system, because eyes derived from *miracle-gro* or *jolly green giant* clones in the EGUF/*hid* assay [43] described below were enlarged.

#### General tracheal cell undergrowth (2B)

In roughly 500 lines, homozygous mutant cells in mosaic animals were small or absent, and mutant terminal cells when present were small with few or no branches (Figure 3C). Most of these lines presumably carried mutations in general cell growth (“house-keeping”) genes, but we suspected a subset might carry mutations in genes specifically required for tracheal cell growth. Indeed, we found that clones mutant for the tracheal master regulator *trachealess,* resembled clones homozygous mutant for the housekeeping gene *glutamyl-prolyl-tRNA synthetase (* Figure S1). To distinguish tracheal-specific from more general cell growth mutations, we tested the ∼500 tracheal undergrowth mutations in the developing eye. We used the EGUF/*hid* system [43] in which forced expression of a cell death gene in the eye imaginal disc eliminates wild type cells, so adult eyes develop almost entirely from clones of homozygous mutant cells. Nearly all (>99%) of the tracheal cell undergrowth mutations failed to rescue eye development, resulting in adults lacking eyes or with grossly undersized eyes. This substantiated that these mutations affected more general cell growth genes, and the lines were discarded. However, two tracheal undergrowth mutants, *lotus* (312) (Figure 3C) and *etiolated* (1736), formed eyes of normal size and shape, demonstrating that they are tracheal-selective growth mutations. Other aspects of the *lotus* phenotype are detailed below.

*sprout* (574) caused a general reduction of tracheal cell size, as well as a growth defect in the EGUF/Hid assay. However, it was distinguished from all the general undergrowth mutations described above by the ability of the small mutant terminal cells to branch extensively, like a bonsai plant (Figure 3D).

#### Selective tracheal cell undergrowth (2C)

Two mutations, *cincher* (773) and *corset* (897), caused a growth defect selective for dorsal trunk cells: homozygous mutant dorsal trunk cells were less than half normal size, whereas mutant terminal cell clones were unaffected (Figure 3E). This selectivity contrasts with that of the ∼500 house-keeping mutants, many of which caused their most pronounced effects on terminal cells, presumably because of the dramatic growth and branching these cells undergo during larval life. Thus, *cincher* and *corset* are growth genes required in dorsal trunk but dispensable in terminal cells.

### Class 3: branch number, pattern, size, and shape mutants

These mutations affected the number of terminal branches, and in some cases also the position at which new branches bud from the parental branches. Many also affected the shape of the buds and mature branches. Mutations caused different but characteristic spectrums of defects so that, for example, mutations that reduced terminal cell branching to a similar extent could reproducibly give rise to terminal cells of very different morphology, such as short and thick versus elongate and wispy.

#### Terminal branch pruning (3A)

**Figure 4:**
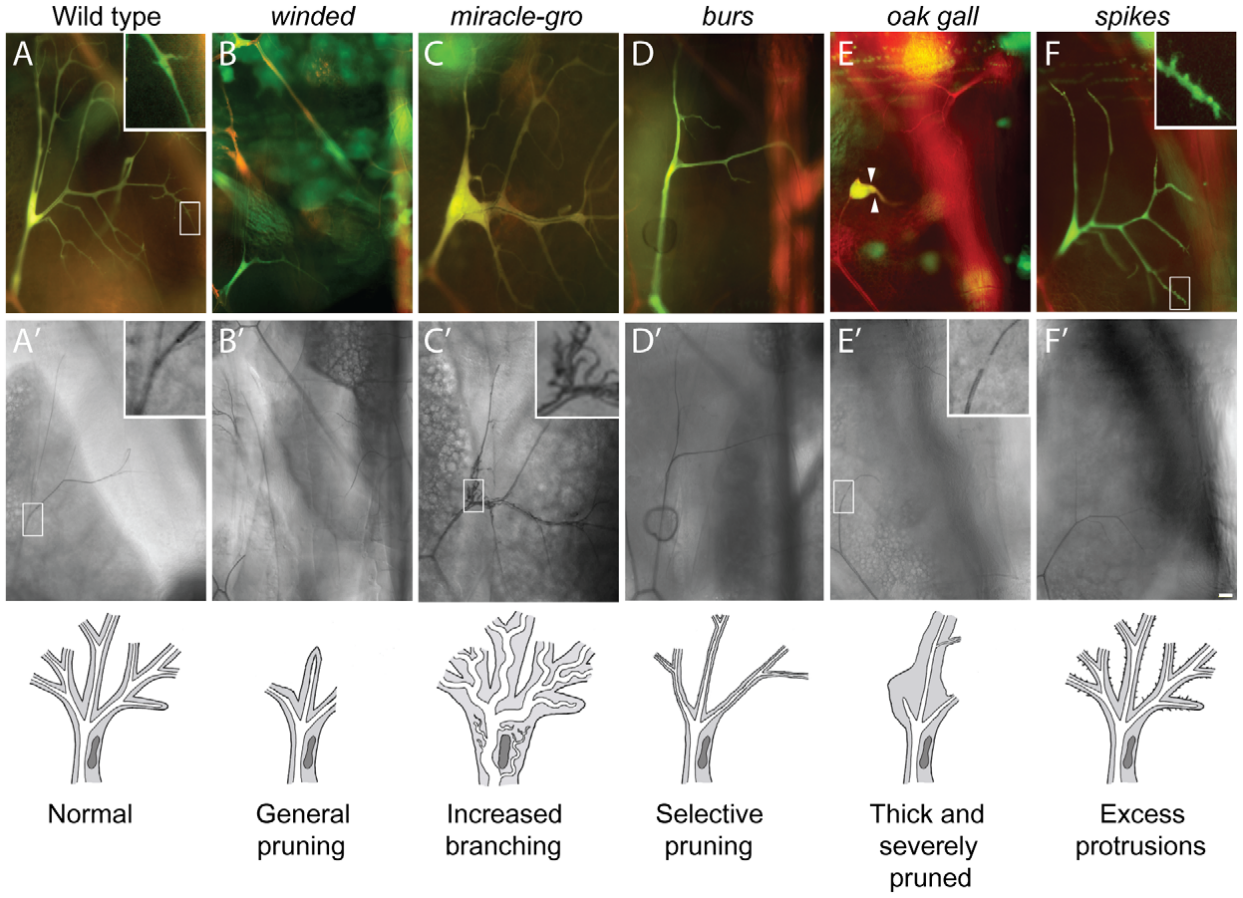
Terminal cell branching mutants. Fluorescence (A–F) and brightfield (A'–F') images of homozygous terminal cell clones (DsRED^+^, GFP^+^ so appear yellow in A–F) of the mutations indicated, with schematics of the phenotypes shown below. Open boxes, area enlarged in insets. (A, A') Control wild type clone. There are dozens of terminal branches (A), and each mature branch contains a single, continuous gas-filled lumen (A'). New terminal branches arise from filopodial growth cones (A, inset). (B, B') *winded^1508^* clone. Note absence of terminal branches. (C, C') *miracle-gro^1483^* clone. Note enlarged branches and multiple convoluted seamless tubes in enlarged soma (C', inset). (D, D') *burs^1139^* clone. Note presence of first generation terminal branches but absence of most second and all subsequent generations. (E, E') *oak gall^696^* clone. Note all but one terminal branch is missing, and remaining branch is short and stout (arrowheads). Another phenotype is the tiny gap in the gas-filled lumen at or near the position where autocellular and subcellular tubes connect in terminal cell (E', inset; compare to inset in A'). (F, F') *spikes^773^* clone. Note excess filopodia arising from terminal branches (F, inset) but normal or slightly reduced numbers of mature terminal branches (F'). Bar, 20 µm.

We identified ∼40 mutants in which terminal cell clones had fewer branches than normal but were distinct from the general cell growth genes described above because mutant dorsal trunk cells were of normal size and morphology. This phenotype is similar to that of *blistered (pruned),* the canonical terminal branching gene [14],[44]. *winded* alleles (613,1227,1375,1508) showed severe pruning defects, like *blistered* null alleles: mutant terminal cells had few branches, and any residual branches were typically thin and wispy and lacked subcellular tubes (Figure 4A, 4B). Other severe pruning mutants were *paltry* and *topiary*. Mutations in most other genes of this class showed more modest pruning defects (e.g. *lopped, truncated*), comparable to *blistered* partial loss of function alleles.

#### Excess branching (3B)

Although many mutations that reduced terminal branching were identified, mutations that increased branching were rare. Indeed, the only mutations that increased branching were the *miracle-gro* alleles described above, which also increased cell size. Although some extra branches observed in *miracle-gro* terminal cells might simply be terminal branches that are normally present but bigger than normal and hence easier to detect, there clearly were also extra branches not present in wild type, such as those in proximal positions of *miracle-gro* terminal cells. In many cases, these extra seamless tubes coursed through the soma of the terminal cell, giving it a striking multiple lumen phenotype (Figure 4C′, inset).

#### Branch pattern and shape alterations (3C)

Eight mutations affected terminal cell branch pattern or shape and included mutations that caused selective pruning (category 3C1, e.g. *burs*), thick and severely pruned branches (3C2, e.g. *lotus*), and excess protrusions (3C3, e.g. *spikes*), as detailed below.

*burs* (942, 1139) mutant terminal cells ramified much less extensively than wild type, but the branching defect appears selective for side branches and possibly other later rounds of terminal branching (Figure 4D). Likewise, in the complementation group *denuded* (PC213, 1520) the first terminal branches appeared largely normal, although sometimes thickened, but subsequent branches were absent or severely compromised: small, unbranched, and with a narrow bore tube. These mutants demonstrate genetic differences between early and later rounds of terminal branching.

Terminal cells mutant for *lotus* (312), *conjoined* (356), and *oak gall* (696) were severely pruned, in the most extreme cases with just a single significant branch, and the remaining branches were unusually thick (Figure 4E). However, lumens within the thickened branches were of normal diameter.

*spikes* (735) mutant terminal cells had variable numbers of cytoplasmic protrusions emanating along the length of terminal branches (Figure 4F, inset), protrusions that in wild type are typically restricted to the growing tip and sites of lateral sprouting (Figure 4A, inset). Most of the excess protrusions, however, were short and nonproductive as *spikes* mutant terminal cells had slightly fewer mature terminal branches than normal.

### Class 4: tube formation, number, position, and shape mutants

**Figure 5:**
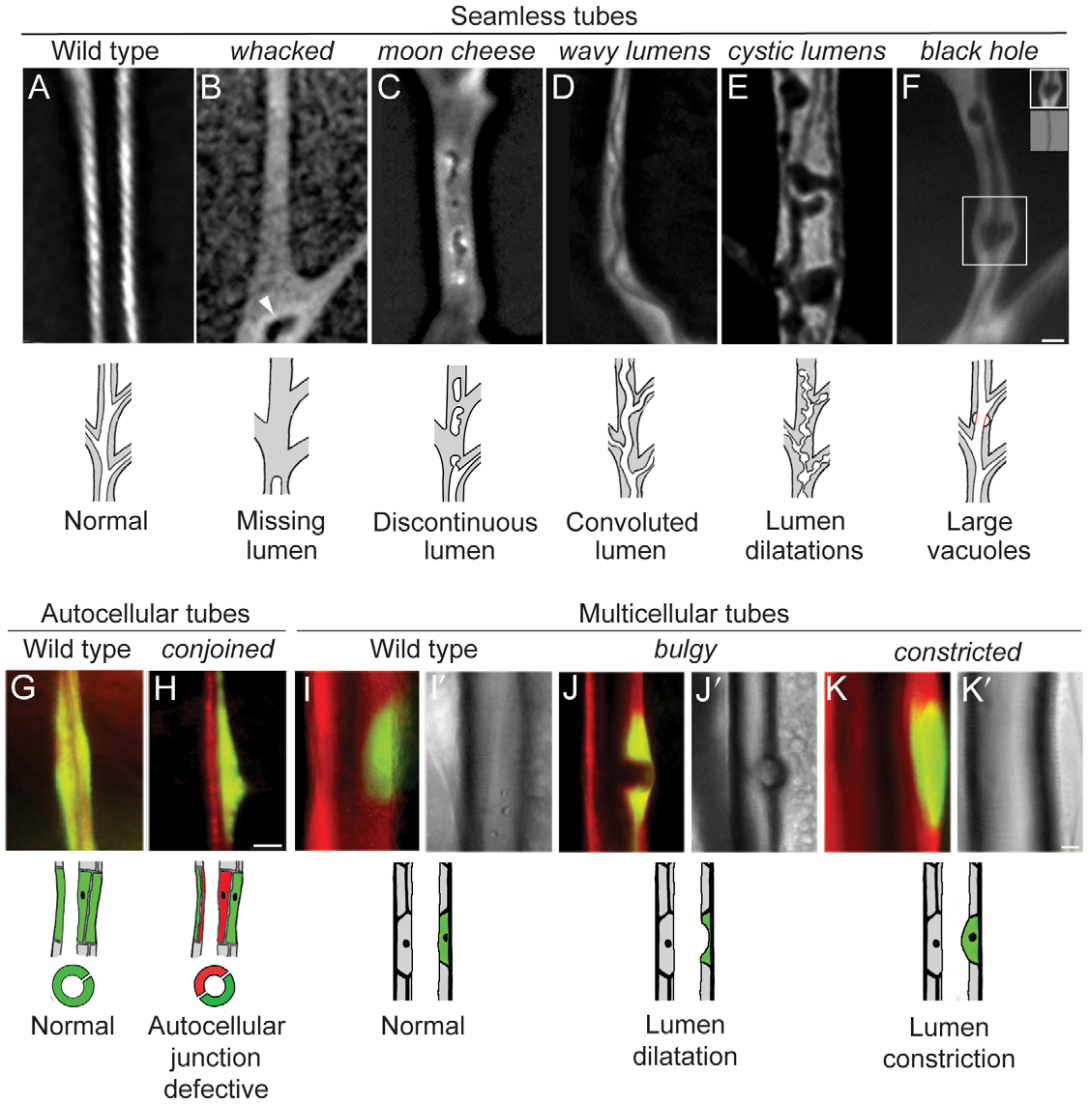
Tubulogenesis mutants. Fluorescence photomicrographs of control wild type (A, G, I) and homozygous mutant (B–F, H, J, K) clones in seamless, autocellular, and multicellular tracheal tubes in third instar larvae. Schematics of the phenotypes are diagrammed below. Clones are marked with GFP (white in A–F, green in G–K) and all tracheal cells with DsRED (red in G–K); brightfield images in I'–K' show air-filled lumens of multicellular tubes. (A) Wild type control clone in seamless tube. (B) *whacked^220^* clone. Note most of the lumen is missing and the terminus of the residual lumen (arrowhead) is dilated and irregularly shaped. (C) *moon cheese^1524^* clone. (D) *wavy lumens^894^* clone. (E) *cystic lumens^1243^* clone. (F) *black hole^538^* clone. The regions where the lumen appears to be dilated (e.g., boxed area, upper inset) are actually regions in which a vacuole, which can be distinguished from the lumen by its accumulation of lumGFP (not shown), intimately surrounds a lumen of normal diameter (lower inset, brightfield view of boxed area). The vacuole is outlined in red in schematic. (G) Wild type control clone in autocellular tube. The single marked cell (GFP^+^, green) surrounds the lumen, sealed by an autocellular junction. (H) *conjoined^356^* clone. The mutant cell (GFP^+^, green) does not form an autocellular junction but instead forms the lumen by making intercellular junctions with a heterozygous cell (DsRED^+^, red). (I) Wild type control clone in dorsal trunk, a multicellular tube. (J) *bulgy^636^* clone. Lumen bulges outward into mutant cell, forming a local dilatation. (K) *constricted^960^* clone. Lumen constricts inward at site of mutant cell by ∼7% relative to the neighboring, fully wild type dorsal trunk segments. Bar, 5 µm (A–F), 10 µm (G,H), 10 µm (I–K).

**Figure 6:**
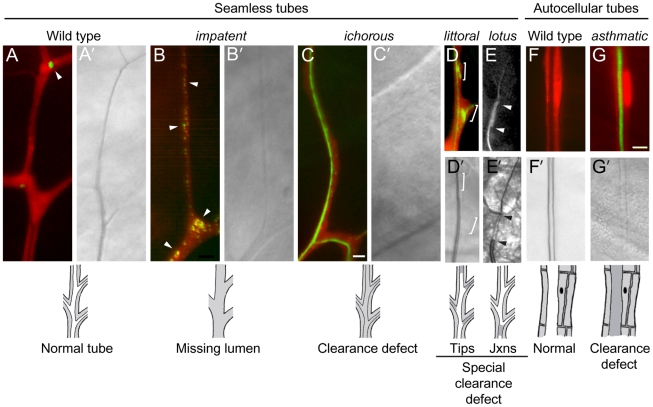
Lumen clearance and gas-filling mutants. Fluorescence (A–G) and bright field micrographs (A'–G') of control wild type (A, F) and homozygous mutant clones (B–E, G) in seamless and autocellular tracheal tubes as indicated. Clones are labeled with cytoplasmic DsRed (red) and also express lumGFP (green), a secreted form of GFP; the fluorescence micrographs (A–G) are DsRed/lumGFP merged images, except for E, which shows only the lumGFP channel (white). Lumen defects are diagrammed below, with air-filled lumens in white and matrix-filled lumens and tracheal cell cytoplasm in grey. (A, A') Wild type control terminal cell. lumGFP has been cleared from the mature, gas-filled lumen (A'). The only lum-GFP visible is small puncta in the cytoplasm at the tip (A, arrowhead). (B, B') *impatent^1757^* clone. This is a mutant, like those described in Figure 5, in which the seamless lumen is missing (B'): lumGFP is detected only in puncta (B, arrowheads), presumably aberrant intermediates in lumen formation, distributed in the soma and along the lumenless terminal branch. (C, C') *ichorous^206^* clone. Although no mature, gas-filled lumen is detected by brightfield optics (C') as in *impatent* mutant cells, a lumen has formed–just not cleared–as shown by luminal lumGFP staining (C). (D, D') *littoral^762^* clone. A specialized clearance defect: the central terminal branch forms a normal gas-filled lumen but the tips of growing side branches (brackets) contain a lumen that has not cleared (D') and remains loaded with lumGFP (green, D). (E, E') *lotus^312^* clone. Another specialized clearance defect, restricted to the junction between (arrowheads) the base of the branch (connection with stalk cell) and the seamless tube. The fluorescence signal in E above and below the arrowheads is autofluorescence of the cuticle, not lumGFP. (F, F') Control wild type autocellular tube. (H, H') *asthmatic^1530^* clone. Lumen is difficult to detect (G') because it remains filled with luminal matrix and lumGFP (green, G). Bar, 5 µm (in C, A–E), 10 µm (F,G).

These mutations prevented lumen formation, or altered the number, placement, or shape of the lumens that formed. Most of these mutations did not affect all tracheal tubes, but rather structurally distinct subsets of tubes. We start with a description of mutations that affect the seamless tubes of terminal branches (see Figure 1B; Figure 5A–5F; Figure 6B).

#### Seamless tube defects (4A)

These mutations caused defects in seamless tubes including missing and discontinuous lumens (category 4A1, e.g. *impatent*), convoluted lumens (4A2, e.g. *wavy lumens*), multiple convoluted lumens (4A3, e.g. *tendrils*), lumen dilation and other irregularities (4A4, e.g. *cystic lumens*), and large vacuoles (4A5, e.g. *black hole*), as detailed below.

We recovered ∼25 lines in which gas-filled lumens were not detected in terminal cell clones by brightfield microscopy. Some of these mutants are defective in lumen formation as assessed by a secreted GFP reporter (lum-GFP), whereas others form lumens that are difficult to detect by brightfield microscopy because they remain filled with matrix (see below). The three alleles of *impatent* (1472, 1490, 1757) lacked or had seriously compromised seamless tubes, whereas other aspects of terminal branches appeared normal and lumens of other branches were unaffected. In *impatent* mutant terminal cells, the lumGFP reporter accumulated in large puncta, up to 1–2 um in diameter, located mostly in the cell body (Figure 6B). These were probably enlarged vesicles representing trapped or aberrant intermediates in lumen formation.

Some of the other mutations in this class displayed a more complex spectrum of defects. *whacked* mutations (PC24, 220) eliminated the distal portions of seamless tubes, with the lumen typically terminating prematurely in an irregularly-shaped local dilatation **(** Figure 5B). *moon cheese* (1524) and *carbuncle* (804) caused a variable discontinuous lumen phenotype in which regions along the terminal branch were missing lumen but flanked by regions containing blind-ended and irregularly-shaped lumen (Figure 5C). These mutations also caused a fully penetrant large vacuole phenotype (see below).

In *wavy lumens* (894) and *wobbly lumens* (BG13), terminal cell lumens formed but followed a convoluted path through the cytoplasm (Figure 5D). Similar convoluted lumens were seen in *miracle-gro* mutant terminal cells and cells exposed to hypoxia [45]. However, the convoluted lumens of *wavy lumens* and *wobbly lumens* were not associated with excessive terminal cell growth and branching as in *miracle-gro* mutants and under hypoxia: *wobbly lumens* terminal cells were of normal size and *wavy lumens* terminal cells were mildly pruned.

*miracle-gro* mutations caused multiple convoluted lumens in the soma of mutant terminal cells, along with the extra growth and branching described above. Eight additional mutants were identified that had multiple, disorganized lumens but fewer branches than normal. Four compose the complementation group *tendrils* [46], and *vine* (512) defines another locus whose phenotype was remarkably similar to *tendrils* except that it began to manifest a day or so earlier in development. *creeper*, *braided* and *ivy* had similar but less penetrant phenotypes.

Several mutants had irregular but gas-filled tubes. As described above, mutations in *whacked* had irregularly shaped, and often prematurely terminated tubes. *cystic lumens* (*1243*) caused areas of lumen dilation and constriction (Figure 5E) and occasional gas-filling defects. The tube morphology defects in these mutants most closely resemble those previously reported for larger tubes (dorsal trunk) in which the secretion or modification of chitin is affected [47], and more recently by mutation of receptor tyrosine phosphatase activity [48].

Other mutations caused large cytoplasmic vesicles that excluded cytoplasmic GFP in mutant tracheal cells. In *black hole* (538) mutant cells, the vacuoles were centered over gas-filled lumens, suggesting that transport into the lumenal space is defective (Figure 5F). *black hole* and *dark matter* (1417) caused vacuole accumulation specifically in tracheal terminal cells, whereas *moon cheese* (1524) and *carbuncle* (804) caused vacuole accumulation in all tracheal cells. For *moon cheese* and *carbuncle*, a secreted form of GFP (lum-GFP) accumulated to high levels within mutant cells, particularly within the vacuoles, suggesting a defect in trafficking of secreted proteins. *moon cheese* and *carbuncle* also caused a variable discontinuous tube defect described above.

#### Autocellular tube defects (4B)

*lotus* (312), *conjoined* (356), *oak gall* (696) interfered variably with the formation of tubes sealed by autocellular junctions (Figure 1B): mutant dorsal branch cells appeared either to completely avoid contributing to autocellular tubes, forming exclusively intercellular rather than autocellular junctions (Figure 5G, 5H), or contributed only minimally with most of the cell body protruding basally away from an otherwise smooth epithelial tube. Dorsal trunk clones also protruded basally, although still formed intercellular junctions (not shown). These mutations demonstrate that autocellular junctions and tubes are genetically distinct from intercellular junctions and multicellular tubes (Figure 1B).

#### Multicellular tube defects (4C)

These mutations caused defects in multicellular tubes, including lumen dilation (category 4C1, e.g. *bulgy*) and lumen constriction (4C2, e.g. *short of breath*), as detailed below.

*small potatoes* (1113, 1166, 1694), *bulgy* (636), and *balloon* (1448) caused subtle outward bulges in the lumen of dorsal trunk tubes (Figure 5I, 5J). Bulges occurred at the sites of clones, even single cell clones, hence these mutations identify cell autonomous regulators of multicellular tube diameter.

Eight mutations caused the opposite phenotype: dorsal trunk cells mutant for *short of breath* (5 alleles), *dyspneic (1348, 1359)*, and *constricted* (960) caused lumenal constrictions at the sites of mutant cells (Figure 5K), reminiscent of the recently described mutant *stenosis* [49].

### Class 5: lumen clearance/gas-filling mutants

We expected that some mutants from the screen that appeared to lack lumens would instead be lumen clearance and gas-filling mutants in which the lumen was present but difficult to detect by brightfield optics because it remained filled with matrix, which has a similar refractive index as the surrounding cytoplasm. To identify such mutants, lines from the screen that appeared under brightfield optics to lack lumens were subjected to a secondary screen using a transgene expressing a fusion protein containing the signal peptide of p23 [50] linked to GFP. The fusion protein (lumenal-GFP or lumGFP) was designed to transit the secretory pathway, as it does in mammalian cells [50], entering the tracheal lumen and remaining there to mark the lumen of mutants that affect lumenal clearance, such as *ichorous* and *asthmatic* (Figure 6C). By contrast, no lumenal accumulation of lumGFP was observed in mutants such as *impatent* described above that lack or have seriously compromised lumens (Figure 6B), or in wild type control clones because lumGFP is cleared from the lumen during the normal lumenal maturation process (Figure 6A). The only lumGFP that remained in *impatent* mutant clones was the large puncta already described (Section 4A1), and the only lumGFP detected in control clones was the rare puncta near branch tips or the junctions between branches (Figure 6A). In mutations such as *scrub* (659), lumGFP was not detected in either matrix-filled lumens or cytoplasmic puncta (data not shown); these mutations might alter lumenal targeting such that lumGFP is secreted from other positions in the cell so does not accumulate intracellularly.

#### Seamless tube clearance defects (5A)

These mutations caused liquid clearance defects in all seamless tubes (category 5A1, e.g. *asthmatic*), in the tips of seamless tubes (5A2, e.g. *littoral*), or at the junction between seamless and autocellular tubes (5A3, e.g. *lotus*), as detailed below.

Mutants such as *asthmatic* and *ichorous* (Figure 6C) described above, and *stertorous* and *liquid-filled,* were defective in matrix clearance: mutant terminal cells had normal morphology and formed seamless tubes, but the tubes failed to clear and gas-fill. These cells cannot supply oxygen to their targets, and neighboring wild type cells were frequently found invading the region normally supplied by the mutant cell; under normal conditions, terminal cell domains do not overlap [45], similar to neuronal tiling [51].

Seven mutants (*littoral*, *burs, ivy, panting,* 826, 928, 1809) had gas-filling defects that typically affected only new or distal portions of subcellular tubes (Figure 6D). The tip-clearance defects were variable, with some mutant terminal cells more severely affected than others, even within the same mosaic animal. These mutants suggest that the tips of terminal branches have specialized requirements for clearance and gas filling, or that these regions are particularly sensitive to defects in the general machinery.

*lotus*, *oak gall, conjoined*, and *disjointed* mutants showed an exquisitely specific clearance and gas-filling defect: in mutant terminal cells, no gas-filled lumen could be detected connecting the secondary branch tube, which has an autocellular tube, to the terminal branch seamless tubes, which lack junctions and extend throughout the rest of the terminal cell and gas-filled normally (Figure 4E′, Figure 6E and 6E′).

#### Other branches (5B)

Nine mutants representing four complementation groups showed dramatic defects in liquid clearance/gas-filling of tracheal tubes containing autocellular junctions. Two of the complementation groups, *short of breath* and *dyspneic*, were described above, because they also cause defects in dorsal trunk and terminal cells (sections 4C2 and 5A1). *asthmatic* mutations, which caused a strong terminal cell gas-filling defect as noted above, also caused a partially penetrant autocellular tube gas-filling defect (Figure 6F, 6G). *flashflood* (*AP67*) selectively blocked clearance of lateral tracheal trunks, as detected in homozygous third instar larvae.

### Mutation mapping and gene identification reveal new cell biological pathways in tracheal development

To begin to define the molecular functions of tracheal genes identified in the screen, we mapped representative mutations and molecularly identified 14 of the genes (Table 2). Six of the identified genes *(no tc clones-L, no tc clones-R, short of breath, dyspneic, lopped* and *failed fusions*) were previously implicated in tracheal development. However, new functions were revealed for each by our clonal analysis. Two are allelic to canonical tracheal genes in the *branchless*/*breathless* FGF pathway, the *breathless* FGFR itself [26] and *pointed* [11], [59], which we showed are differentially required for competition during tip cell selection [13]. Two others, *short of breath* and *dyspneic*, which our results implicate in lumen clearance and gas-filling and as cell autonomous promoters of tube expansion, are allelic to *krotzkopf verkehrt* (*kkv*) and *knickkopf* (*knk*), chitin synthesis pathway genes previously shown to coordinate the behavior of cells in the tracheal epithelium during tube expansion [53], [54], [60], [61]. Our results demonstrate that chitin synthesis genes also have an unexpected, cell autonomous function in lumen clearance and gas filling of autocellular and seamless tubes. *lopped^784^* is allelic to *fatiga* that encodes *Drosophila* Hif1 prolyl hydroxylase, and appears to be required in terminal cells for normal branching. Previous studies with hypomorphic *fatiga* alleles gave opposite results [56], although new studies indicate that early exposure to hypoxia (mimicked by loss of *fatiga*) result in stunted tracheal development while later exposure stimulates branching [64]. *failed fusions* is allelic to *polychaetoid,* which has been implicated in branch fusion and in tracheal cell intercalation, but our mosaic analysis, along with other new data from our lab, suggest another function for *polychaetoid* in tip cell selection (E. Chao, A.S.G. and M.A.K., unpublished data).

**Table 2:** Molecular identification of tracheal genes.

Comp. Group^1^	Alleles	Map Position	Comp. Test^2^	Mutation [strength^3^]	Flybase Name (gene loc'n)	Protein Function	Extant Tracheal Function^4^	Reference	P/M^5^
*burs*	942 1139	73D1	*TSG101^D9^ TSG101^D10^ TSG101^D18^*	CCC>TCC (P86L) CCT>CTT (P113L)	*TSG101* (73D1)	Homolog of TSG101/VPS23, part of ESCRTI complex in endosome sorting	None	[52]	M
*dyspneic*	1348 1359	3R	*knk* 1	ND	*knickkopf* (85F13)	Chitin synthesis protein, dopamine β-monooxygenase motif	Size and shape of dorsal trunk lumen	[28], [53], [54]	M
*failed fusions*	AZ63 PA14	3R	*pyd^C5^*	ND	*polychaetoid* (85B2-7)	Homolog of ZO-1 junctional MAGUK	Cell intercalation	[37]	M
*jolly green giant*	1149	95D; 95F & 98E; 99A	*TSC1^9834^*	ND	*TSC1* (95E1)	Homolog of tumor suppressor TSC1; putative vesicular transport role	None	[55]	M or P
*lopped*	784	82F	*l(3)82Fe dHph^02255^*	ND	*Hph* (82F7-82F8)	Homolog of Hif1 prolyl hydroxylase, regulator of Hif1α transcription factor	Inhibition of terminal cell branching	[56]	P
*miracle-gro*	338 878 1483 1489	99F; 100B	*lats^XI^ wts^MGH1^*	ND	*warts* (100A5)	MD kinase homolog in *hippo* growth control pathway	None	[57], [58]	M or P
*moon cheese*	1524	64C;D		CAG>TAG (Q85stop)	*membrin* (61C13-64C14)	Homolog of membrin, an ER-Golgi t-snare	None	This study	M
*no terminal cell clones-L*	602 724 788 1118 1187 1476 1684 BN40	*h-th*	*btl^LG18^*	ND TGG>TGA(W275stop) CAG>TAG (Q296stop) CGC>CAC (R863H) TCG>TTG (S912L) [m] CCA>TCA (P487S) CGA>TGA (R402stop) GAG>AAG (E796K) [w] (Ref 10)	*breathless* (70D2)	FGFR, receptor for Branchless-FGF	Primary, secondary and terminal branching	[13], [26]	P
*no terminal cell clones-R*	198 1313	94D; 95A	*pnt^D88^*	ND	*pointed* (94E10-94E13)	Ets-box transcription factor	Secondary and terminal branching	13,59	P
*short of breath*	360 404 483 761 791 1705	82F	*kkv* 1	ND	*krotzkopf verkehrt* (83A1)	Chitin synthase	Size and shape of dorsal trunk lumen	[28], [53], [60], [61]	M
*tendrils*	666 1308 1469 1539	*ru-h*	*rhea^79A^*	CAA>TAA (Q1250stop) AG>AA (splice site) [a] CAG>TAG (Q934stop) CAG>TAG (Q2051stop) [w] (Ref 16)	*rhea* (66D6-66D7)	Homolog of Talin, an integrin/actin cross-linker	None	[46], [62]	M
*vine*	512	89E		GGT>GAT (G297D)	*cctg* (89D6)	Homolog of cct-γ, component of cct/TriC chaperonin	None	This study	M
*whacked*	PC24 220	86 E14; 86E17		(Schottenfeld and Ghabrial, unpublished data)	*whacked* (86E11)	Putative RabGAP	None	This study	M
*winded*	613 227 1375 1508	65F3; 66B10	*cdsA* 1 *cdsA^7^*	ND CTC>TTC (L227F), TGG>AGG (W238R) ND TGG>TAG (W83stop)	*cdsA* (66B7)	Homolog of CDP diglyceride synthetase in PI biosynthesis	None	[63]	M or P

1 Complementation group name.

2 Mutations in known genes that failed to complement tested mutations in complementation group.

3 The relative strengths of the sequenced alleles of each gene were similar unless noted in brackets next to an allele that it was weak [w] or moderate [m] compared to the other, presumed null allele(s), or stronger than the presumed null and likely antimorphic [a].

4 Previously known tracheal function.

5 P, presumptive patterning gene; M, presumptive morphogenesis gene.

The other eight molecularly identified genes had not been previously implicated in tracheal development; indeed, three (*vine*, *moon cheese*, and *whacked*) had not been genetically defined (Table 2). All eight identify new cell biological pathways in tracheal development. *jolly green giant*, which encodes the *Drosophila* ortholog of TSC1 [55], [65], and *miracle-gro* (see below*)* [57], [58] implicate general growth control pathways in tracheal growth and branching. *tendrils*, which is allelic to *rhea* and encodes talin [46], and *vine*, which encodes the *Drosophila* ortholog of CCTgamma, show that talin-dependent integrin adhesion and a component of the TriChaperonin complex are required for maintenance of terminal branches and lumenal organization.

The four other genes implicate membrane and vesicle trafficking genes in tracheal development. Such genes have been speculated to function in tube morphogenesis but few have been genetically identified. *winded*, essential for terminal branching, encodes the *Drosophila* homolog of CdsA [63], an enzyme that converts phosphatidic acid to cds-diacyl glycerol in the production of the membrane lipid, phosphatidyl inositol. *moon cheese*, another terminal branching gene also implicated in lumen continuity, and *burs*, a terminal branching gene selectively required for side branches, encode the *Drosophila* homolog of the ER-Golgi t-SNARE membrin, and TSG101/*erupted*, a component of the ESCRTI complex that sorts endocytic vesicles to the multivesicular body, respectively [52], [66], [67]. *whacked*, which promotes the growth and proper shape of terminal cell lumens, encodes a putative RabGAP (A.S.G. and M.A.K., unpublished data). The identification of membrane lipid and vesicle trafficking genes in terminal branching supports the idea that outgrowth of cellular processes and lumen formation require targeting of apical and basolateral membrane components at a distance from the cell soma. It will be important to determine the number of trafficking pathways involved, how the pathways are activated at the appropriate times and places, and how the identified t-SNARE, Rab-GAP, and ESCRTI component function in the pathways.

Thus, all 14 molecularly characterized genes from the screen reveal new cell biological pathways in tracheal development or new functions for established pathways.

### Most of the identified genes are morphogenesis genes

The identities of the molecularly characterized genes allowed us to assess the success of the screen in identifying morphogenesis effectors. Although it was not possible to unambiguously classify all 14 genes in this way from their sequence alone, eight very likely function as morphogenesis effectors: the vesicle trafficking genes *moon cheese/membrin*, *burs/TSG101,* and *whacked/RabGAP*; the cell junction and cytoskeletal genes *failed fusions/polychaetoid/ZO-1*, *rhea/tendrils/talin*, *vine/cctγ*; and the chitin synthesis genes *short of breath/kkv/chitin synthase* and *dyspneic/knk*. Three others are established patterning genes: the receptor *btl/no-terminal cell clones-L*, the transcription factor *pnt/no terminal cell clones-R*, and the transcription factor regulator *lopped/fatiga/Hif prolyl hydroxylase*. The remaining three are more difficult to categorize because they encode enzymes that likely couple patterning signals to cytoplasmic outgrowth *(winded/(CdsA)* and cell growth (*Tsc1*/*jolly green giant* and *miracle-gro),* as discussed below. Thus, over three-quarters of the identified genes (11 of 14, 79%) appear to be downstream effectors/morphogenesis genes (8 of 14, 57%) or genes that couple patterning signals to morphogenesis (3 of 14, 21%), supporting our hypothesis that systematic clonal analysis is an effective way of identifying such genes.

### A cell growth regulator that also regulates lumen morphogenesis

**Figure 7:**
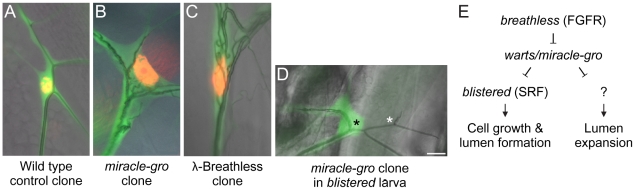
Genetic analysis of terminal cell growth control pathway. (A–D) Close-ups of the soma of third instar larva terminal cell clones of the indicated genotypes. Terminal cell cytoplasm is marked with GFP (green) and nuclei in A–C are marked with nuclear DsRed2 (red). Note that the cell body and nucleus of the *miracle-gro(warts)^388/388^* clone (B) and the clone expressing λ*-*Breathless (C), a constitutively active form of Breathless FGFR, are enlarged with ectopic lumens coursing through the soma. By contrast, the soma of the *miracle-gro(warts)^388/388^* clone in a larva homozygous for *blistered^ l(2)3267^,* a downstream transcription factor in the Breathless pathway (D), is smaller and there are no ectopic lumens (black asterisk). However, the single, truncated lumen of the clone is dilated compared to the truncated lumen of the contralateral control terminal cell (white asterisk). (E) Genetic pathway of terminal cell growth control. Bar, 20 µm.

In addition to identifying new tracheal genes and pathways, the screen suggested new functional connections between pathways. One example came from characterization of the *miracle-gro* cell overgrowth mutations (Section 2A). In terminal cells, not only was the soma enlarged but there were many ectopic seamless tubes coursing through it (Figure 4C, 4C′ and Figure 7B). This phenotype is nearly unique: it is seen otherwise only upon hyperactivation of the Breathless FGFR pathway (Figure 7C). However, *miracle-gro* mutations did not map near *breathless* or any other extant loci in the pathway. Mapping and complementation tests (Table 2) demonstrated that *miracle-gro* is allelic to *warts/lats-1* [57], [58], which encodes a kinase that suppresses cell growth in a well-established general growth control pathway. Thus, loss of a key growth regulator in terminal cells leads not only to excessive cell growth but excessive lumen formation, revealing an unexpected coupling between cell growth control and tubulogenesis.

The striking similarity of the *warts/miracle-gro* loss of function phenotype and the *btl* pathway gain-of-function phenotype suggested that FGF signaling might stimulate terminal cell growth and tubulogenesis by inactivating Warts function. To test this, we sought to define the genetic epistasis relationship between *warts/miracle-gro* and *breathless*-FGFR pathway mutations. Because mutations that disrupt FGF signaling abrogate terminal cell specification [11], [29], it is not possible to generate terminal cells doubly mutant for *warts* and *breathless*, so we examined terminal cells doubly mutant for *warts/miracle-gro* and *blistered*/*pruned/SRF*, the downstream transcription factor in the *breathless* FGFR pathway required for terminal cell growth and branching. Doubly mutant cells were unbranched and small, similar or slightly bigger than *blistered* mutant terminal cells, and with a single lumen in the soma (Figure 7D). However, the lumen diameter was larger than normal and similar in size to those in *warts/miracle-gro* mutant terminal cells. Thus, the cell growth and excessive lumen formation seen in *warts/miracle-gro* mutant terminal cells are dependent on *blistered*, whereas lumen diameter can be modulated independently of *blistered*. This supports a model in which Warts/Miracle-gro functions downstream of Breathless FGFR but upstream of Blistered/SRF in the regulation of terminal cell size and lumen number, and upstream of another, as yet unidentified, transcription factor that controls lumen diameter (Figure 7E).

## Discussion

Our systematic screen for tracheal mutations on the third chromosome identified new tracheal phenotypes and scores of new tracheal genes as well as new functions for established tracheal genes. Molecular identification of 14 of the genes indicates that most of the isolated genes are downstream effectors/morphogenesis genes, an important category of genes substantially underrepresented in previous screens. Several of the identified genes encode proteins involved in vesicle trafficking, implying that such genes are a major class of morphogenesis genes, at least for terminal branching.

Our screen succeeded in identifying morphogenesis genes because (i) it was systematic and extensive, surveying most third chromosome genes, nearly 40% of the genome; (ii) it included a clonal analysis of mutations using a new cell marking method that allowed facile identification of tracheal functions of pleiotropic mutations such as vesicle trafficking and cytoskeletal genes; and (iii) it employed a secondary screen in another tissue (eye) to exclude mutations in general housekeeping genes. Housekeeping genes are a huge class of genes that would have dominated the results of our clonal screen, as they have in previous clonal screens [35]. Exclusion of housekeeping genes also allowed the identification of tracheal-selective growth regulators, which contributed to our discovery of cell growth control as an important new step in branching (see below).

**Figure 8:**
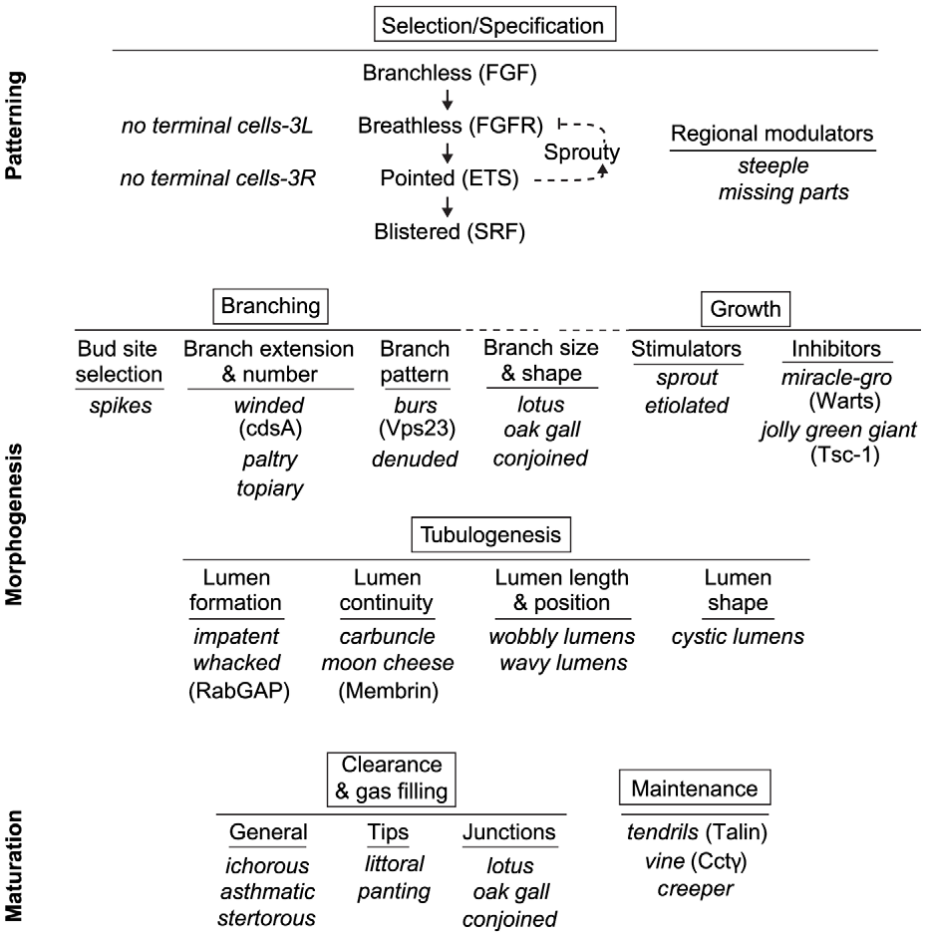
Genetic dissection of terminal branch morphogenesis. The major, genetically separable processes in the terminal branching program are illustrated, in the order in which they occur, along with representative mutations that disrupt them. There is an initial patterning step (Selection/Specification) that selects and specifies the terminal cell, followed by five morphogenesis (Branching, Growth, Tubulogenesis) and maturation (Clearance/Gas-Filling, Maintenance) steps. The steps can be functionally subdivided further by the more specific phenotypes of the mutants shown. Where the molecular identities of the genes are known, the protein products are given (in parentheses) to indicate some of the molecular functions involved in each step. The SRF transcription factor Blistered (Pruned), a key regulator of terminal branching and the last gene in the Selection/Specification step, presumably controls expression of at least some of the downstream morphogenesis and maturation genes including ones involved in growth and tubulogenesis (Figure 7E).

Many of the mutations we identified, particularly in the clonal analysis, affect terminal branching, a morphogenesis process for which little was known beyond the key signaling pathway and transcription factors that control terminal cell selection. The systematic nature of our screen and the distinct phenotypes of terminal branching mutations we identified provide a comprehensive genetic outline of this morphogenetic process (Figure 8). We propose that terminal branching involves an initial cell selection and specification step plus five major morphogenesis processes: branching, growth, tubulogenesis, gas filling, and maintenance. Each of these processes is associated with one or two defining genes that are required quite generally for the process plus additional genes, mutations in which further subdivide each process into distinct morphogenetic steps or reveal additional levels of regulation. Below we discuss each of these processes and the associated genes, highlighting cell growth regulation because its critical role in branching morphogenesis had not been recognized. We return at the end to discuss implications of the results for the corresponding processes in primary and secondary branching.

### Cell selection and specification

The earliest step of cell selection and specification in terminal branching is a well-characterized process that previous genetic studies have shown is controlled by the Branchless FGF pathway (Bnl/Btl/RAS/Pointed) that induces expression of the Blistered/Pruned SRF transcription factor that selects cells at the ends of budding branches for a terminal branching fate [9], [10]. Re-expression of Bnl FGF later in hypoxic tissues is proposed to initiate terminal branch budding, at least in part by activation of the *blistered* SRF transcription complex and its downstream effector genes [14], [45].

Our screen identified third chromosome genes previously implicated in the selection process, but revealed an interesting new aspect of the process because of the novel “no terminal cell clones” phenotype. These turned out to be mutations in *breathless* FGFR and *pointed* [13], and lead to the discovery there is specialization among cells in a budding branch and only the leaders need to receive the Branchless FGF signal. Cells mutant for Breathless FGFR cannot receive the signal, and are relegated to trailing positions, never to be specified a terminal cell.

We also discovered genes (*steeple*, *missing parts*) required for specification of a subset of terminal cells in specific regions or branches. These may encode region-specific enhancers of the Bnl-Btl pathway because sporadic failure of terminal cell formation is seen in animals in which this signaling pathway is partially compromised [11], [13], [34].

### Branch budding and extension

Although the Branchless pathway and Blistered SRF transcription complex are key regulators of branch budding and outgrowth [14], [29], little is known of the signal transduction pathway that connects them or of the downstream effectors. *winded/cdsA mutations* caused a severe, cell autonomous terminal branch pruning phenotype similar to that of *blistered*/SRF null alleles. *winded/cdsA* encodes an enzyme (CDP-diacylglycerol synthase) required for phosphoinositide (PI) synthesis, suggesting that a PI-dependent signaling process, presumably like those involved in other receptor tyrosine kinase (RTK) signaling pathways [68], functions downstream of the Btl RTK in the control of Blistered/SRF and branch budding. Some of the other genes with pruned phenotypes (e.g., *topiary, paltry, truncated)* might encode additional signal transduction components or targets of the SRF transcription factor required for polarized cell growth (see below).

We propose that other branching genes regulate bud site selection and the pattern of branching. *spikes* encodes a negative regulator of bud site selection because small ectopic buds form in mutant terminal cells. One appealing idea is that *spikes* restricts the normal budding response of terminal cells to the sites of maximal induction by Branchless FGF. *TSG101/erupted/burs* and *denuded* regulate branch pattern by promoting lateral and late rounds of terminal branching, perhaps by catalyzing the local disassembly or reorganization of the cytoskeleton within a maturing terminal branch.

One set of genes (*lotus, oak gall, conjoined*) affected branch number but also dramatically altered the size and shape of the remaining branches (see below). These were difficult to categorize purely as branching genes or growth regulators, so we place them in a special class at the boundary between those categories because they share features of both. They may function as integrators of branch outgrowth and size control signals.

### Growth regulation

A new aspect of the tracheal developmental program highlighted by the mutants is cell size and growth regulation. Outgrowth of terminal branches requires not only chemoattractant signaling to induce and guide migration, but synthesis of cellular and membrane components to support cytoplasmic outgrowth. Terminal cells mutant for *glutamyl-prolyl-tRNA synthetase* or hundreds of other presumptive house keeping genes failed to form and extend terminal branch buds. Many such growth-promoting genes were presumably among those identified in a previous clonal screen for tracheal mutations [35], but because there are many such mutations and their phenotypes are non-specific (small, sick, or missing cells) and difficult to distinguish from genes simply required for cell viability, it was hard to evaluate their developmental significance.

Three types of data argue that growth control is an integral part of the tracheal developmental program. First, clonal analysis of the master regulator *trachealess* in terminal cells gave a similar phenotype (Figure S1), implying that terminal cell growth is a process actively regulated by Trachealess. *etiolated* is a particularly interesting mutant of this class because it resembled *trachealess* not just in its clonal phenotype in terminal cells, but in its tracheal specificity. Second, we obtained mutations in two canonical growth suppressor genes, *warts*/*miracle-gro* and *Tsc1*/*jolly green giant*, which gave the opposite phenotype: terminal branches and terminal cells were overgrown with particularly large somas that in *warts/miracle-gro* mutant cells contained multiple seamless tubes passing through them. This shows that a general growth regulator controls not only cell size but tubulogenesis, an essential step in the tracheal developmental program. Third, the phenotype of *warts/miracle-gro* mutant terminal cells is very similar to that of activated Btl, and genetic epistasis experiments suggest that *warts/miracle-gro* functions downstream of, and is negatively regulated by, *btl* FGFR but upstream of *blistered* SRF (Figure 7E).

*sprout* is the most intriguing undergrowth mutant because it was the only one that formed small but normally patterned terminal branches. *sprout* cleanly decouples branch size from branch budding and outgrowth, so we propose it is a key gene in branch size control.

Three other genes, *lotus*, *oak gall,* and *conjoined,* also function in branch size control, but in a different way. In mutant cells, branches were much thicker and more variable than normal, but the diameter of the seamless tubes that form within them were normal. We propose that these genes function in the size control pathway by regulating the distribution of plasma membrane or other cell constituents among branches. When this process fails, branches become thicker and fewer in number.

Most of the undergrowth mutations and all of the overgrowth mutations affected not only terminal cells but other tracheal cell types and cells outside the tracheal system, implying that the affected genes encode general growth regulators. However, many undergrowth mutations had their most extreme effects on terminal cells, presumably because they are larger and grow more than other cells. But two mutations, *cincher* and *corset*, affected the growth of dorsal trunk cells and spared terminal cells. Thus, the growth control programs of these tracheal cell types are genetically separable. Because terminal cell growth appears to be controlled primarily or exclusively by Bnl-Btl signaling and operates selectively under hypoxic conditions, conditions that arrest the growth of most other cell types, *cincher* and *corset* might identify specific regulators or components of aerobic growth pathways or other general growth processes dispensable in terminal cells.

### Tubulogenesis genes

The striking phenotype of *impatent* mutant terminal branches, branches that superficially appear normal but lack air-filled tubes and hence are nonfunctional, leads us to propose that *impatent* encodes a key regulator or component of terminal branch lumen formation. We further propose that lumen shape is governed by *cystic lumens,* perhaps in conjunction with *whacked*, mutations in which result in irregularly-shaped lumens, and that lumen length and position are controlled by *wobbly lumens* and *wavy lumens,* mutations that cause long and convoluted lumens. Long and convoluted lumens are also seen in terminal cells under hypoxic conditions and other conditions that cause excessive branchless FGF pathway activity and/or terminal cell growth (e.g. *warts*/*miracle-gro*), so an appealing model is that these conditions and this signaling pathway inhibit the activity of *wobbly lumens* and *wavy lumens*, which themselves function to restrict the length or the position of terminal branch lumens.

Lumen continuity requires *carbuncle* and *membrin*/*moon cheese*, suggesting that lumens of seamless tubes are made piecemeal and these genes promote their connection. We also identified four genes (*disjoined*, *lotus*, *conjoined*, and *oak gall*) required to make functional connections between seamless tubes and the adjacent, architecturally distinct autocellular tubes that form by wrapping. The short lumenal gap in these mutant terminal cells may result from a failure to connect the tubes or a structural defect that prevents clearance of the connection.

### Lumen clearance and gas filling genes

For tracheal branches to become functional, the lumenal matrix must be cleared and replaced by gas, the molecular composition of which is unknown. *ichorous and asthmatic* are required for clearance and gas filling of most or all terminal branches, and *littoral* and *panting* and others are required to clear the tips of terminal branches. Recent studies highlight the importance of secretion into the lumen and subsequent endocytosis of lumenal matrix and liquid during tube expansion and air-filling of large multicellular tracheal tubes [16], [69]; the genes identified in our screen may mediate related processes in seamless tubes.

### Maintenance genes

These genes maintain the elaborate shape and structure of terminal branches under mechanical stress such as muscle contraction. In the mutant *rhea*/*tendrils/talin* [46], terminal branches begin to form normally but branches break down and their lumens retract as the larva begins to move and the developing branches are subjected to the stress of stretch. The phenotypes of *cctgamma*/*vine*, *creeper*, *braided*, and *ivy* are similar to *tendrils*, suggesting that they function in the same integrin/talin cell adhesion and cytoskeletal support system. For example, the *cctgamma*/*vine* chaperonin may facilitate the folding or assembly of talin or some other component in the support system, an appealing hypothesis given that CCT chaperonins have been shown in other systems to mediate the folding of cytoskeletal proteins [70].

These maintenance genes emphasize the importance of analyzing the onset and evolution of a mutant phenotype when elucidating gene function, because similar phenotypes can arise from early developmental aberrations or later defects in maintenance. Other elaborate cell types and organs likely also require maintenance genes. For example, mutations in mouse Dlg5 perturb delivery of adherens complex proteins to the plasma membrane of brain and kidney epithelial tubes, resulting in cyst formation [71]. Many such structural maintenance genes are expected to function late in life so would be missed in typical developmental screens. A major effort should be aimed at their identification and isolation because of their importance in medicine and disease.

### Coordination and coupling of morphogenesis processes

How are the genetically separable terminal branch morphogenesis processes described above coordinated and controlled in time and space? An important part of this control and coordination almost certainly involves the Branchless FGF pathway. Expression of both the ligand *branchless* and the receptor *breathless* are induced by hypoxia during larval life, and terminal cell outgrowth and branching are stimulated and directed to hypoxic cells by local production of Branchless FGF [56]. One way Bnl-Btl signaling stimulates branching is likely through transcriptional induction of morphogenesis genes via modification and activation of the Blistered /SRF transcription complex. In other systems, the SRF transcription complex has been shown to be regulated by the actin cytoskeleton and to regulate cytoskeletal genes [72], and such genes are almost certainly required for growth of the actin-rich terminal branch buds. It will be important to determine which of the identified morphogenesis genes are regulated by Branchless signaling and SRF, and to identify the full set of downstream targets by transcriptional profiling.

Because Branchless functions as a chemoattractant, it must also provide a spatial cue that guides terminal branch outgrowth. One appealing idea is that the ligand-bound Breathless FGFR at the surface of the terminal cell generates a spatial cue that directs polarized growth of cytoplasmic extensions toward hypoxic, FGF-secreting cells. Such a spatial cue could be used to direct vesicular traffic to the growing ends of terminal cell extensions, both for polarized growth of the new branch and construction of a lumen within it. The spatial cue might be a modified membrane PI because PI signaling functions downstream in many RTK signaling pathways [68] and can regulate vesicle trafficking [73] and tubulogenesis [74], and mutations in the PI synthesis gene *cdsA*/*winded* severely abrogated terminal branching.

Although Branchless-Btl signaling likely controls and coordinates many of the events in terminal branch morphogenesis, it is unlikely to be the sole control and coordination mechanism because not all of the morphogenesis events occur at the same time and place. Lumen formation (tubulogenesis) occurs after cytoplasmic outgrowth, and lumen maturation including clearance and gas filling occur even later, in some cases days after the lumen has formed. Likewise, branch and lumen maintenance are late steps in the process. Although there may be delays built into some of the effector pathways downstream of Breathless to stagger its effects, other factors likely also contribute to the timing and spatial organization of the events. For example, ecdysone signaling may gate the timing of lumen clearance and gas filling, and there are presumably cell intrinsic cues that direct transport vesicles carrying integrins and other basolateral markers to the plasma membrane of growing buds, and vesicles carrying apical markers and lumenal components to internal positions.

One surprising finding of our clonal analysis of known tracheal genes was that terminal cell clones of the tracheal master regulator *trachealess* gave a pruned phenotype. This implies that *trachealess* is required not only for its well established role in the initiation of tracheal development [23], [24], but also for much later steps in the developmental program such as terminal cell growth and branching. Perhaps it functions in conjunction with SRF and other cell type and stage-specific transcription factors in the program to impart tracheal specificity in the control of downstream effector genes, as shown for the C. elegans pharyngeal master regulator *pha-4* [75].

### Primary and secondary branch morphogenesis genes

We identified a number of genes required for proper formation of the larger branches of the tracheal system that form earlier than terminal branches and from which they arise. For example, we identified mutants required for proper shape of multicellular tubes, including mutations that cause tracheal dilatations (*small potatoes*, *bulgy*, balloon) and others that cause local constriction of multicellular and autocellular tubes (*kkv*/*short of breath*, *knk*/*dyspneic*, *constricted*). An especially intriguing set of genes (*lotus*, *conjoined*, *oak gall)* are those required to form autocellular junctions and lumens. These may encode specialized components or regulators of autocellular junctions and tubes, such as proteins required for a cell to wrap on or seal to itself.

Although we identified some primary and secondary branch morphogenesis genes, there was a surprising paucity of such genes relative to the large number of terminal cell branching genes identified; a similarly skewed distribution obtained in a second chromosome screen, if the large number of putative housekeeping genes is excluded [35]). Although it is possible that morphogenesis of these larger branches requires fewer genes, more likely such genes were just not as efficiently identified in our screen. One reason is that some such genes only show a tube phenotype when most or all cells in the branch are mutant, as with *breathless* (Figure 2B) and *grainyhead* mutations [76]. Another reason is that perdurance of maternally expressed gene products likely obscures early functions of some genes. Finally, terminal cells have an elaborate structure that may make them more sensitive to mutations and makes phenotypes easier to detect.

### The number of genes required to build the tracheal system

Our systematic genetic dissection of an organogenesis process, including a clonal analysis to identify tracheal genes with pleiotropic functions, allows an estimate of the number of genes required to build an organ–an important question not just for developmental biology but for medicine and tissue engineering. Because we identified ∼70 tracheal genes on the third chromosome (Table 1 and Table S3), which represents ∼40% of the genome, the full genome likely contains roughly two hundred genes required to construct the larval tracheal system. This almost certainly represents a lower limit because our screen did not achieve full saturation and, as described above, the screen would miss essential embryonic genes required non-cell autonomously and genes with a significant maternal contribution.

Genomic profiling of developing and mature organs indicates that there are hundreds of differentially expressed genes among different organs, and genetic profiling to identify downstream genes of organ master regulators such as the *C. elegans* pharyngeal regulator *pha-4* [77], the mouse pancreas regulator Pdx1 [78], [79], and the *Drosophila* tracheal regulator Trachealess (E. Chao and M.A.K. unpublished data), suggests that there are 110–240 genes dependent on the master regulator for expression, at least at certain stages of development. Although it is not known how many of these downstream genes are required for organ morphogenesis, or what fraction of organ morphogenesis genes are both selectively expressed and downstream targets of organ master regulators, these genomic results are in line with the estimate from our genetic studies that organ morphogenesis programs, even ones for relatively simple organs like the *Drosophila* tracheal system, are likely to involve several hundred genes. The approach used here, involving a clonal analysis in the tracheal system of all mutations that do not survive late enough in development as homozygotes to assess their tracheal function, has begun to be extended to the other major chromosomes to identify the rest of the tracheal morphogenesis program [7], [35] (Metzstein M. and M.A.K., unpublished data); most of the identified mutations fit with the genetic scheme described here, with the exception of a novel set of mutations on the second chromosome that compromise terminal branch mutual avoidance and spacing [35]. The clonal approach could easily be adapted to other organs to systematically dissect additional organogenesis programs.

## Materials and Methods

### Drosophila strains

*D. melanogaster* strains used in the screen and meiotic mapping experiments were: (1) *btl*-GAL4, UAS-GFP; *Pr*, Hs-*hid*/TM3Sb, Tub-GAL80, (2) a newly isogenized *btl*-GAL4, UAS-GFP; FRT2A,FRT82B, (3) *y w* FLP^122^; *btl*-GAL4, UAS-DsRED; FRT82B *cu* UASi-GFPhp/TM6B, (4) *y w* FLP^122^; *btl*-GAL4, UAS-DsRED; UASi-GFPhp *th st* FRT2A/MKRS (5) *y w ey*-FLP; cell-lethal, GMR-*hid* FRT2A/MKRS, (6) *y w ey*-FLP; FRT82B, cell-lethal, GMR-*hid*/MKRS, (7) y w FLP122; *breathless*-GAL4, UAS-lumGFP, UAS-DsRED; FRT82B TubGal80, (8) y w FLP122; *breathless*-GAL4, UAS-lumGFP, UAS-DsRED; TubGal80 FRT2A; (9) a newly isogenized *ru h th st cu sr e ca;* (10) *ru h th st cu sr e Pr ca*/TM6B. Other strains were: FRT2A, FRT82B (from Trudi Schüpbach); Hs-*hid* (on chromosome III; from Ruth Lehman), onto which *Pr* was recombined; TM3Sb, Tub-GAL80 (from Stefan Luschnig); and strains used in complementation tests and deficiency mapping experiments (see Table S3). All other strains, except the mutants isolated here, have been described (http://flybase.bio.indiana.edu) and are available from http://flystocks.bio.indiana.edu.

### Vector and transgene construction

UAS-DsRed. This Gal4-dependent DsRed transgene was constructed by inserting a 0.7 kb Kpn I-Xba I restriction fragment containing the DsRed coding sequences from pDsRed (Clontech) between the corresponding sites of the vector pUAST [80]. The resultant plasmid, pUAST-DsRed, was used to establish transgenic lines on the X, second, and third chromosome by P element mediated transformation of *w^1118^* embryos. The second chromosome insertion (line 5A) was recombined with *breathless*-GAL4 and used here.

pUASTi. This P element vector for generating RNAi transgenes was constructed by PCR amplification *(*primers Xho+trh-intron F, Kpn+trh-intron B; see Table S2 for primer sequences) and TA cloning of the 221 bp third intron from the *trachealess* gene into the vector, pCRII-TOPO (Invitrogen). The intron fragment was then excised with Xho I and Kpn I and inserted at those sites in pUAST. Note that the *trachealess* intron contains an Eco RI site, leaving Bgl II, Not I and Xho I as the only unique restriction sites 5′ of the *trachealess* intron, and Kpn I and Xba I as the only unique sites 3′ of the *trachealess* intron. To generate RNAi constructs, a ∼500 bp fragment from the gene of interest is inserted in the forward orientation just upstream of the intron, and in the reverse orientation downstream of the intron, as described below for UAS-GFP(RNAi). Gal4-driven expression of this transgene results in tissue specific transcription of the self-complementary RNA, which is predicted to form a double-stranded “hairpin” conformation that initiates the RNAi response.

UAS-GFP(RNAi). This Gal4-dependent GFP(RNAi) transgene was created by PCR amplification (primers Not-GFP-F, Xho-GFP-R) and insertion of an ∼500 bp fragment of GFP (in the forward, sense orientation) between the Not I and Xho I sites upstream of the intron in pUASTi, and amplification of the same fragment (primers Xba-GFP-F, Kpn-GFP-R) and insertion (in the reverse orientation) between the Kpn I and Xba I sites downstream of the intron, to create plasmid pUASTi-GFP(RNAi). Transgenic flies were generated as above, and insertions were identified on all major chromosomes. For this study, an insertion on 3L (insertion B) was recombined onto *ru h th st* FRT2A (from Stefan Luschnig) to generate the UASi-GFPhp *th st* FRT2A chromosome, and an insertion on 3R (insertion 4A) was recombined onto FRT82B *cu sr e ca* (from S. Luschnig) to generate the FRT82B *cu* UASi-GFPhp chromosome.

UAS-lumGFP. This Gal4-dependent transgene expressing secreted (lumenal or “lum”) GFP was constructed by inserting an Nhe I-Kpn I restriction fragment with the GFP coding sequence from plum-GFP [50] between the Xba I and Kpn I sites of pBS-KS (Stratagene), and then subcloning the Not I/Kpn I lum-GFP fragment into those same sites in the pUAST vector. Transgenic flies were generated as above, and second and third chromosome insertions were recovered. A second chromosome insertion was recombined onto a *breathless*-GAL4 bearing second chromosome for use here.

### Mutagenesis

A standard F3 EMS mutagenesis screen was performed (Figure 1C). Strains used were homozygous for *breathless*-GAL4 [81] and UAS-GFP transgenes on chromosome II. Males homozygous for an isogenized FRT2A, FRT82B chromosome III were fed 25 mM EMS as described [82] and mass mated to *breathless*-GAL4, UAS-GFP; Pr, Hs-*hid*/TM3, Sb, Tub-GAL80 virgin females. F1 males were each mated to two virgins of the genotype used in the P cross. After five days, parents were removed from the F1 cross and on days five and six F2 larvae were heated to 38°C for 1.5 hours to induce Hs-*hid* and eliminate animals carrying that chromosome. In the few cases (∼2–3%) where animals carrying *Pr*, Hs-*hid* survived, virgins of the appropriate genotype were selected to generate a stock of FRT2A, FRT82B*/TM3, Sb, Tub-GAL80 (*, newly induced mutation). If animals homozygous for the treated third chromosome (non-Sb) were not detected in the F3 or subsequent generations, a lethal mutation was assumed to be present. Two hundred mutagenized chromosomes were assayed in three small-scale pilot screens, and 4100 mutagenized chromosomes were assayed in a final large-scale screen.

### F3 screen

Sibling F2 flies (described above) were allowed to mate and were brooded to produce two clutches of F3 individuals, one-quarter of which should be homozygous for the mutagenized third chromosome. The first F3 brood (after five days) was used to maintain the stock and assess for presence of a lethal mutation; the second F3 brood (at 12 days) was screened for tracheal phenotypes (see below). If less than four pairs of flies were obtained in the F2 generation, screening was postponed for a generation. For tracheal phenotype screening, F3 larvae were washed out of their food vials with distilled water and examined under an M2 Zeiss or a Leica fluorescence stereomicroscope. Homozygous third instar larvae were identified by tracheal expression of GFP, and tracheal morphology was analyzed; at least three homozygous larvae were examined for each mutant line. Animals that appeared to have tracheal defects were heat-killed (70–75°C for 3–5 s), mounted in 50% glycerol and examined under a Zeiss Axioplan 2 compound fluorescence microscope. Lines in which a tracheal defect was detected were retested to confirm the phenotype. If no reproducible phenotype was found, the line was discarded. Mutant lines that did not give rise to viable homozygous third instar animals were analyzed in genetic mosaics as follows.

### Genetic mosaic screen

Males from the mutant stock established in the F3 screen were crossed to *y, w,* FLP^122^; *breathless*-GAL4, UAS-DsRED; UASi-GFPhp, *th, st*, FRT2A/MKRS virgin females, and to *y, w,* FLP^122^; *breathless*-GAL4, UAS-DsRED;FRT82B, *cu*, UASi-GFPhp/ TM6B virgin females, to test mutants on 3L or 3R, respectively. For each arm (3L and 3R) of every mutant stock, a cross with 20–40 pair matings was done. Embryos (0–4 hr old) were heat-treated as above for 0.75–1 hr to induce FLP-mediated recombination; animals 2 hrs old or less at the time of heat treatment typically do not survive. Crosses were maintained at 25°C for five days and then mosaic animals were examined under a fluorescence stereomicroscope. All tracheal cells are marked by expression of UAS-DsRED; mutant tracheal cells also express GFP. This GFP marking of mutant cells was achieved by inducing recombination between a chromosome arm carrying the mutation of interest and the homologous chromosome arm carrying UASi-GFPhp (see above). Daughter cells homozygous for the mutagenized chromosome arm lack the GFP(RNAi) transgene and thus express GFP. Animals of the correct genotype were selected, heat killed, and analyzed for tracheal defects as above. Under these clone induction conditions, ∼60±8 (mean +/- SEM) tracheal clones were generated per animal (n = 5 animals). Among dorsal branch clones (n = 127 clones), 50% appeared to be composed of a single cell, 37% of two cells, 10% of three cells, and 3% of four or five cells.

### EGUF/HID secondary screen

Mutations that caused undergrowth defects in tracheal clones were tested for growth defects in eye development using the EGUF/HID technique that generates eye imaginal discs composed exclusively of mitotic clones of a single genotype [43]. Males from mutant lines were crossed to virgin females of genotype *y, w, ey*-FLP; *, GMR-*hid* FRT2A/MKRS, or *y, w, ey*-FLP; FRT82B, *, GMR-*hid*/MKRS, where * is the undergrowth mutation. Adult progeny lacking Sb (carried on both of the balancer chromosomes used) were scored for eye size. Mutations unable to support normal eye development were presumed to affect general cell growth and viability genes (“housekeeping” genes) and were discarded.

### Lumenal GFP secondary screen

Mutants with no detectable lumen under brightfield optics were tested for presence of liquid-filled or discontinuous lumens using the lumGFP transgene described above, which expresses GFP with a signal peptide that we found accumulates in liquid-filled tracheal lumens but is not detectable in normal, gas-filled lumens. Males from the mutant stocks were crossed to *y w* FLP^122^; *breathless*-GAL4, UAS-lumGFP; TubGal80 FRT2A/MKRS or *y w* FLP^122^; *breathless*-GAL4, UAS-lumGFP; FRT82B TubGal80/MKRS virgin females, and mosaic analysis was carried out as described above in the Genetic Mosaic Screen.

### Quantitative analysis of phenotypes

Effect of *miracle-gro^338^* on cell size was determined using ImageJ software to measure the maximal cross-sectional area in a stack of 2D optical sections through the soma of mutant and wild type control terminal cell clones (see Figure 3). Effect on dorsal trunk lumen diameter was determined by comparing lumen diameter between a section of tube containing a single mutant cell and the average diameter of the immediately anterior and posterior regions containing no mutant cells.

### Mutation mapping and gene identification

Initial mapping was carried out by complementation tests against a panel of chromosomal deficiencies spanning the third chromosome, and by meiotic recombination mapping using visible recessive markers *ru h th st cu sr e ca*. Fine scale mapping was carried out using available single nucleotide polymorphisms (SNPs) [83], [84] and new ones specifically identified in this study, in conjunction with complementation tests with chromosomes carrying small, molecularly characterized deletions. The affected gene in the mapped interval was then identified by sequencing candidate genes and comparing their sequences to those in the isogenized parental chromosome to reveal new EMS-induced nonsense mutations or other mutations predicted to compromise gene function, and by complementation tests with extant mutations in the interval.

### *miracle gro/warts* epistasis analysis

To compare the *miracle gro/warts* terminal cell phenotype to that of activated Breathless FGFR, the MARCM system [42] was used to generate marked (GFP^+^) clones of tracheal cells expressing λ-Breathless [85], a constitutively dimerized form of the protein, and marked cells at terminal cell positions were examined using a compound fluorescence microscope. To determine the genetic epistasis relationship between *miracle-gro/warts* and *blistered/pruned/SRF*, a downstream transcription factor in the Breathless pathway, virgins of the genotype *y, w,* hsFLP122; *bs^l(2)3267^*, btl-Gal4, UAS-GFP/CyO; FRT82B *cu*, UAS-GFP(RNAi) were crossed to males of the genotype *bs^l(2)3267^*, btl-Gal4, UAS-GFP/CyO; FRT82B *miracle gro/warts^338^*/MKRS. Mutant animals homozygous for *bs^l(2)3267^* were identified by the strong pruned phenotype of unmarked (GFP^-^) terminal cells, and marked (GFP^+^) *wts^338^* mutant terminal cell clones in these animals were examined and scored as above.

## Supporting Information

Figure S1 Tracheal terminal cell clones mutant for *trachealess* resemble those for the house keeping gene *Aats-gln*. Fluorescence (A–D) and brightfield (A',D') images of larval dorsal branch terminal cell (A,D) or dorsal trunk (B,C) clones (DsRED^+^, GFP^+^; yellow) homozygous mutant for the house keeping gene glutamine aminoacyl tRNA synthetase (*Aats-gln^05461^*; A, C) or the tracheal master regulator *trachealess (trh^10512^*; D). Mutant DB terminal cells are small and lack terminal branches (asterisks in A,D) and air-filled lumens (dashed ovals in A',D'). Control DB terminal cells (DsRED^+^, GFP^-^; red) are shown at left in the images. Homozygous *Aats-gln^05461^* mutant dorsal trunk tracheal cells (yellow cells in B) are smaller than control wild-type dorsal trunk cells (yellow cells in C). Bars in A (for A,D) and C (for B,C), 50 µm.
(TIF)

Table S1 Molecularly identified tracheal genes. Previously identified Drosophila genes with defined tracheal phenotypes. Each of the genes has been assigned as either a presumptive tracheal patterning (P) or morphogenesis (M) gene.
(DOC)

Table S2 Polymerase chain reaction (PCR) primers. Primers were used to generate the modified pUAST vector, pUASTi, and the GFP(RNAi) construct, pUASTi-GFPhp.
(DOC)

Table S3 Additional tracheal morphogenesis mutants. Genes that were identified in the screen but represented by a single allele, and for which the molecular identity of the gene remains unknown. Abbreviations and gene mapping methods are given in the footnotes of Table 1. The estimate of 70 tracheal genes identified in our screen includes the 58 named loci in Table 1 plus the 12 loci in this table (PC146, 137, 198, 826, 889, 928, 1055, 1106, 1631, 1663, 1801) in which a mapped lethal mutation was identified by deficiency mapping (see “Map Position”). However, these mapped lethal mutations may not be in all cases the mutation responsible for the tracheal phenotype.
(DOC)
