# Supplementary figures and images for: A Systematic Screen for Tube Morphogenesis and Branching Genes in the Drosophila Tracheal System
Source: PLoS Genet. 2011 Jul 7;7(7):e1002087. doi: 10.1371/journal.pgen.1002087 (PMC3131284; doi:10.1371/journal.pgen.1002087)

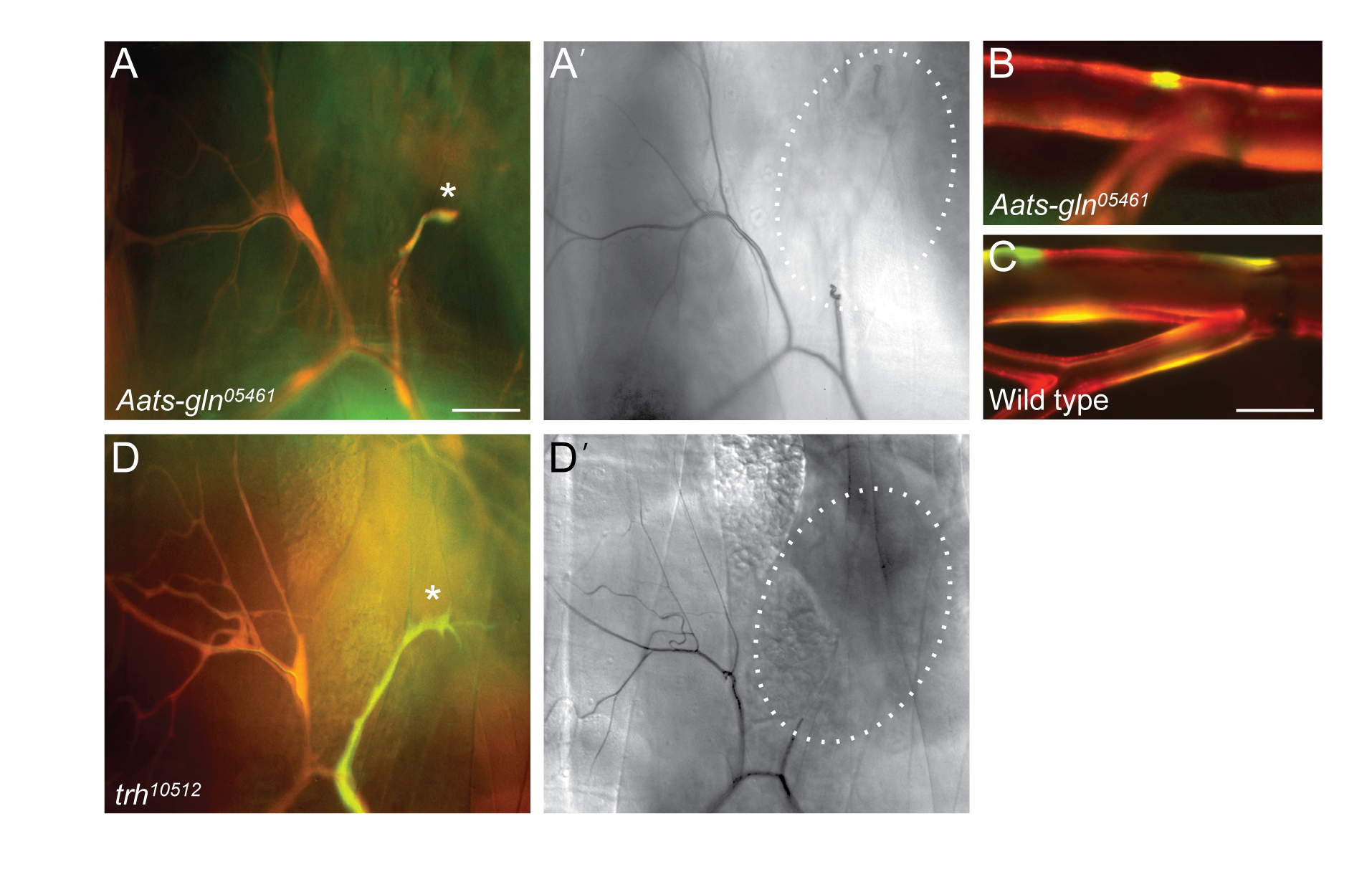

Supplement: Figure S1 — Tracheal terminal cell clones mutant for trachealess resemble those for the house keeping gene Aats-gln. Fluorescence (A–D) and brightfield (A',D') images of larval dorsal branch terminal cell (A,D) or dorsal trunk (B,C) clones (DsRED+, GFP+; yellow) homozygous mutant for the house keeping gene glutamine aminoacyl tRNA synthetase (Aats-gln05461; A, C) or the tracheal master regulator trachealess (trh10512; D). Mutant DB terminal cells are small and lack terminal branches (asterisks in A,D) and air-filled lumens (dashed ovals in A',D'). Control DB terminal cells (DsRED+, GFP-; red) are shown at left in the images. Homozygous Aats-gln05461 mutant dorsal trunk tracheal cells (yellow cells in B) are smaller than control wild-type dorsal trunk cells (yellow cells in C). Bars in A (for A,D) and C (for B,C), 50 µm. (TIF) [file pgen.1002087.s001.tif]
