# Supplementary material for: A Systematic Screen for Tube Morphogenesis and Branching Genes in the Drosophila Tracheal System
Source: PLoS Genet. 2011 Jul 7;7(7):e1002087. doi: 10.1371/journal.pgen.1002087 (PMC3131284; doi:10.1371/journal.pgen.1002087)
Supplement: Table S1 — Molecularly identified tracheal genes. Previously identified Drosophila genes with defined tracheal phenotypes. Each of the genes has been assigned as either a presumptive tracheal patterning (P) or morphogenesis (M) gene. (DOC) [file pgen.1002087.s002.doc]

Table S1. Molecularly identified tracheal genes

| **SIGNALING PATHWAYS** | | | | |
| --- | --- | --- | --- | --- |
| **Gene** | **Encoded product** | **Tracheal phenotype** | Refs | **P / M**^1^ |
| **FGF Pathway** |  |  |  |  |
| *anterior open (aop)/yan* | ETS-box transcriptional repressor | Increased *btl* mRNA expression in the embryonic trachea | [1] | P |
| *branchless (bnl)* | Ligand | No tracheal branching/migration | [2] | P |
| *breathless (btl)* | Receptor | No tracheal branching/migration | [3,4,5,6] | P |
| *corkscrew (csw)* | Protein phosphatase | Branching defects | [7] | P |
| *dally-like* | Glypican | Primary branching defects | [8] | P |
| *Heparan sulfate 6-O-sulfotransferase (dHS6ST)* | dHS6ST | Reduced primary branching | [9] | P |
| *pointed (pnt)* | ETS-box transcription factor | Defective secondary and tertiary branching | [10,11] | P |
| *sprouty (sty)* | Inhibitor of RTK signaling | Extra terminal cells and tracheoles | [12] | P |
| *stumps (sms)/dof/hbk* | Adaptor protein | No tracheal branching/migration | [13,14,15] | P |
| *sugarless* | UDP- D-glucose dehydrogenase | Variable primary branching defects | [16] | P |
| *sulfateless* | Heparan sulfate N-deacetylase/N-sulfotransferase | Variable primary branching defects | [16] | P |
|  |  |  |  |  |
| **TGF-β Pathway** |  |  |  |  |
| *Mothers against DPP (Mad)* | SMAD transcription factor | Defective dorsal/ganglionic branching | [17] | P |
| *nejire (nej)* | CBP- cytoplasmic adaptor | Defective ganglionic branching | [18] | P |
| *punt (put)* | TGFβ receptor type II | Defective dorsal/ganglionic branching | [19,20] | P |
| *thickveins (tkv)* | TGFβ receptor type I | Defective dorsal/ganglionic branching | [21] | P |
|  |  |  |  |  |
| **Wnt Pathway** |  |  |  |  |
| *armadillo (arm)* | β-Catenin | No DT or visceral branch | [22,23] | P |
| *frizzled (fz)* | Receptor | No DT | [23] | P |
| *frizzled-2 (fz2)* | Receptor | No DT | [23] | P |
| *pangolin (pan)/dTCF* | HMG-box transcription factor | No Dt or visceral branch | [23] | P |
| *porcupine (por)* | ER-localized protein | No DT or visceral branch | [23] | P |
| *wingless (wg)* | Ligand | No DT or LT | [22,23] | P |
|  |  |  |  |  |
| **EGF Pathway** |  |  |  |  |
| *faint little ball (flb)/Egfr* | Receptor | Defective invagination of tracheal placode | [20] | P |
| *rhomboid* | Serine peptidase in ligand processing | Defective invagination of tracheal placode | [20] | P |
| *spitz (spi)* | TGF-α type ligand | Defective invagination of tracheal placode | [20] | P |
| *Star* | Ligand processing | Defective invagination of tracheal placode | [20] | P |
| *Ptp4E* | Receptor tyrosine phosphatase | Lumenal defects | [24] | P |
| *Ptp10D* | Receptor tyrosine phosphatase | Lumenal defects | [24] | P |
|  |  |  |  |  |
| **Hedgehog Pathway** |  |  |  |  |
| *hedgehog (hh)* | Ligand | Branch migration defects | [25] | P |
| *patched (ptc)* | Receptor | Branch migration defects | [25] | P |
|  |  |  |  |  |
| **Notch Pathway** |  |  |  |  |
| *Delta (Dl)* | Ligand | Altered tip cell number and specification | [26,27] | P |
| *Notch (N)* | Receptor | Altered tip cell number and specification | [26,27] | P |
|  |  |  |  |  |
| **Slit/Robo Pathway** |  |  |  |  |
| *roundabout-1* | Receptor | Branch misrouting | [28] | P |
| *roundabout-2* | Receptor | Branch misrouting | [28] | P |
| *rhomboid-3* | Ligand processing | Branch misrouting | [29] | P |
| *slit* | Ligand | Branch misrouting | [28] | P |
| *vilse* | Rac/Cdc42 GAP | Branch misrouting | [30] | P |
|  |  |  |  |  |
| **Jak/Stat Pathway** |  |  |  |  |
| *domeless (dome)* | Receptor | No tracheal specification | [31,32] | P |
| *signal transducing adaptor molecule (stam)* | Janus kinase (Jak)-associated signal-transducing adaptor molecule | Cell movement defect in air sacs | [33] | P |
| *stat* | Transcription factor | No tracheal specification | [31,32] | P |
|  |  |  |  |  |
| **Other Signaling Genes** |  |  |  |  |
| *abnormal wing discs (awd)* | Nucleoside diphosphate kinase | Complex branching defects | [34] | P |
| *arl3* | Small GTPase | Tube fusion | [35] | P |
| *Rac* | Small GTPase | Reduced primary branch migration | [36] | P |
| *rhoA* | Small GTPase | Fusion defects | [37] | P |
| *Sar1* | Small GTPase | DT tube expansion and clearing defects | [38] | P |
| *src42A* | Kinase | Defective adherins junction remodeling | [39] | P |
| *src64B* | Kinase | Defective adherins junction remodeling | [39] | P |
|  |  |  |  |  |
| **TRANSCRIPTION FACTORS** | | | | |
| **Gene** | **Encoded product** | **Tracheal phenotype** | Refs | **P / M** |
| *apontic (apt)/trachea defective (tdf)* | bZIP transcription factor | Defects in invagination and primary branching | [40] | P |
| *B-lymphocyte-inducing maturation protein (Blimp1)* | Zinc finger and SET/PR domain containing transcription factor | Dorsal trunk tube diameter | [41] | P |
| *blistered (bs)/pruned/SRF* | Transcription factor | No terminal branching | [42] | P |
| *Dhr78* | Hormone receptor/transcription factor | Liquid clearance/gas-filling | [43] | P |
| *dysfusion (dys)* | bHLH PAS transcription factor | Fusion defects | [44] | P |
| *elbow* | Zinc finger transcription factor | Defective sorting of cells into primary branches | [45] | P |
| *extramacrochaetae (emc)* | Transcriptional co-repressor | Defective primary branching | [46] | P |
| *escargot (esg)* | Zinc finger transcriptional repressor | Defective fusion cell specification | [47,48] | P |
| *grainyhead* | GFH-CP2-type transcription factor | Convoluted lumen | [49] | P |
| *hairy* | Transcriptional repressor | Patterning and lumen size | [50] | P |
| *hunchback* | Zinc finger transcription factor | Fusion defects | [51] | P |
| *jing* | Transcription factor | Tracheal cell death and patterning defects | [52] | P |
| *knirps (kni)* | Zinc finger transcription factor | Dorsal branch cells become dorsal trunk | [53] | P |
| *knirps-like* | Zinc finger transcription factor | Dorsal branch cells become dorsal trunk | [53] | P |
| *MBF* | Transcriptional cofactor | Defective tracheal specification | [54] | P |
| *Mrtf/dMal* | Transcription factor | No terminal branching | [55,56] | P |
| *noc* | Zinc finger transcription factor | Defective sorting of cells into primary branches | [45] | P |
| *pebbled (peb)/hindsight (hnt)* | Transcription factor | Defective taenidia and lumen size | [57] | P |
| *ribbon (rib)* | BTB & pipsqueak domain transcripton factor | Failed branch migration | [58,59] | P |
| *similar (sima)* | bHLH PAS transcription factor, Hifa | Terminal branching defects | [60,61] | P |
| *spalt* | Zinc finger transcription factor | Dorsal trunk cells become dorsal branch | [62,63] | P |
| *stripe (sr)* | Transcription factor | Migration defects | [64] | P |
| *tango (tgo)* | bHLH PAS transcription factor | Branching defects | [65,66] | P |
| *trachealess (trh)* | bHLH PAS transcription factor | Failure of tracheal specification | [67,68] | P |
| *tramtrack (ttk)* | BTB/POZ Zinc finger transcription factor | Fusion cell specification, intercalation | [69] | P |
| *unplugged (unpg)* | Homeodomain transcription factor | Defective branching of CNS trachea | [70] | P |
| *ventral veins lacking (vvl)/drifter (dfr)* | POU-homeodomain transcription factor | Defective tracheal specification | [71,72,73,74] | P |
|  |  |  |  |  |
| **OTHER** | | | | |
| **Gene** | **Encoded product** | **Tracheal phenotype** | Refs | **P / M** |
| *adrift* | Novel nuclear protein | CNS tracheal pathfinding defects | [75] | ? |
| *ATPa* | Sodium/potassium ATPase | Tube size | [76] | M |
| *archipelago* | F-box protein | Fusion cell specification defects | [77] | P |
| *capricious (cap)* | LR protein | Required for fusion of DTa and DTp | [78] | M |
| *congested-like trachea (colt)* | Carnitine antiporter | Required for liquid clearance | [79] | M |
| *Daam* | Formin | Taenidial patterning defects | [80] | M |
| *DE-cadherin/shotgun (shg)* | E-cadherin | Defective fusion cell specification | [48] | M |
| *dumpy (dp)* | ZP domain protein | Lumen defects | [81] | M |
| *von Hippel-Lindau (Vhl)* | Ubiquitin ligase | Complex branching defects | [82,83] | ? |
| *fear of intimacy (foi)* | Zinc ion transporter | Fusion defects | [84] | ? |
| *headcase (hdc)* | PolyQ protein | Ectopic branching defects | [85] | ? |
| *Hsp60C* | Chaperonin | Defective liquid clearance | [86] | M |
| *klarsicht (klar)* | Microtubule based transport | Lumen size and morphology | [50] | M |
| *knickkopf (knk)/gnarled* | Dopamine β-monooxygenase | Tube expansion defect | [87,88,89] | M |
| *krotzkopf verkehrt (kkv)* | Chitin synthase | Tube expansion defect | [87,88,89] | M |
| *Lachesin (Lac)/bulbous* | Ig superfamily member | Tube size and barrier defects | [90] | ? |
| *Lamin (Lam)/misguided* | Nuclear lamin | Terminal cell guidance | [91] | M |
| *megatrachea* | Claudin | Tube size and barrier defects | [92] | M |
| *Myosin heavy chain* | Myosin heavy chain | Under-represented at the distal tip of airsacs in mosaic experiments | [33] | M |
| *MMP1* | Matrix metalloprotease | Dorsal trunk breaks | [93] | ? |
| *mummy (mmy)/ cystic* | UDP-N-acetylglucosamine diphosphorylase | Tube size and barrier defects | [87,94] | M |
| *nervana-2 (nrv2)/ectatic* | Sodium/potassium ATPase | Tube size and barrier defects | [76,95] | M |
| *ninjurin* | Molecular function unknown | no loss of function data (eliminate?) | [96] | ? |
| *pickpocket* | Sodium channel | Clearance/air-filling | [97] | M |
| *piopio (pio)* | ZP domain protein | Cells break away from epithelium | [81] | M |
| *polished rice (pri)* | 4 tiny peptides | Tube expansion | [98] | ? |
| *pollux* | RabGAP | Airfilling/liquid clearance | [99] | M |
| *polychaetoid (pyd)* | ZO-1 homolog | Failed fusions, intercalation defects | [100] | M |
| *retroactive (rtv)* | Molecular function unknown | Tube expansion defects | [101] | ? |
| *serpentine (serp)* | Chitin deactylase | Tube length and tortuosity | [102,103] | M |
| *shortstop (shot)* | Spectroplakin | Fusion cell lumen defects | [37] | M |
| *sinuous* | Claudin | Tube size and barrier defects | [104] | M |
| *tartan* | LR protein | DT fusion defects | [78] | M |
| *varicose* | MAGUK domain septate jxn protein | Elongated dorsal DT tubes | [104] | M |
| *vermiform* | Chitin deacetylase | Tube length and tortuosity | [102,103] | M |
| *wurst* | Novel | Tube matrix clearance defect | [105] | ? |
| *γ-COP* | Vesicle transport/coat protein | Tube expansion defect | [38] | M |
|  |  |  |  |  |

^1^P, presumptive patterning gene; M, presumptive morphogenesis gene.

## **References**

1. Ohshiro T, Emori Y, Saigo K (2002) Ligand-dependent activation of breathless FGF receptor gene in Drosophila developing trachea. Mech Dev 114: 3-11.

2. Sutherland D, Samakovlis C, Krasnow MA (1996) branchless encodes a Drosophila FGF homolog that controls tracheal cell migration and the pattern of branching. Cell 87: 1091-1101.

3. Klambt C, Glazer L, Shilo BZ (1992) breathless, a Drosophila FGF receptor homolog, is essential for migration of tracheal and specific midline glial cells. Genes Dev 6: 1668-1678.

4. Lee T, Hacohen N, Krasnow M, Montell DJ (1996) Regulated Breathless receptor tyrosine kinase activity required to pattern cell migration and branching in the Drosophila tracheal system. Genes Dev 10: 2912-2921.

5. Reichman-Fried M, Shilo BZ (1995) Breathless, a Drosophila FGF receptor homolog, is required for the onset of tracheal cell migration and tracheole formation. Mech Dev 52: 265-273.

6. Ghabrial AS, Krasnow MA (2006) Social interactions among epithelial cells during tracheal branching morphogenesis. Nature 441: 746-749.

7. Perkins LA, Johnson MR, Melnick MB, Perrimon N (1996) The nonreceptor protein tyrosine phosphatase corkscrew functions in multiple receptor tyrosine kinase pathways in Drosophila. Dev Biol 180: 63-81.

8. Yan D, Lin X (2007) Drosophila glypican Dally-like acts in FGF-receiving cells to modulate FGF signaling during tracheal morphogenesis. Dev Biol 312: 203-216.

9. Kamimura K, Fujise M, Villa F, Izumi S, Habuchi H, et al. (2001) Drosophila heparan sulfate 6-O-sulfotransferase (dHS6ST) gene. Structure, expression, and function in the formation of the tracheal system. J Biol Chem 276: 17014-17021.

10. Klambt C (1993) The Drosophila gene pointed encodes two ETS-like proteins which are involved in the development of the midline glial cells. Development 117: 163-176.

11. Scholz H, Deatrick J, Klaes A, Klambt C (1993) Genetic dissection of pointed, a Drosophila gene encoding two ETS-related proteins. Genetics 135: 455-468.

12. Hacohen N, Kramer S, Sutherland D, Hiromi Y, Krasnow MA (1998) sprouty encodes a novel antagonist of FGF signaling that patterns apical branching of the Drosophila airways. Cell 92: 253-263.

13. Michelson AM, Gisselbrecht S, Buff E, Skeath JB (1998) Heartbroken is a specific downstream mediator of FGF receptor signalling in Drosophila. Development 125: 4379-4389.

14. Imam F, Sutherland D, Huang W, Krasnow MA (1999) stumps, a Drosophila gene required for fibroblast growth factor (FGF)-directed migrations of tracheal and mesodermal cells. Genetics 152: 307-318.

15. Vincent S, Wilson R, Coelho C, Affolter M, Leptin M (1998) The Drosophila protein Dof is specifically required for FGF signaling. Mol Cell 2: 515-525.

16. Lin X, Buff EM, Perrimon N, Michelson AM (1999) Heparan sulfate proteoglycans are essential for FGF receptor signaling during Drosophila embryonic development. Development 126: 3715-3723.

17. Steneberg P, Hemphala J, Samakovlis C (1999) Dpp and Notch specify the fusion cell fate in the dorsal branches of the Drosophila trachea. Mech Dev 87: 153-163.

18. Takaesu NT, Johnson AN, Sultani OH, Newfeld SJ (2002) Combinatorial signaling by an unconventional Wg pathway and the Dpp pathway requires Nejire (CBP/p300) to regulate dpp expression in posterior tracheal branches. Dev Biol 247: 225-236.

19. Vincent S, Ruberte E, Grieder NC, Chen CK, Haerry T, et al. (1997) DPP controls tracheal cell migration along the dorsoventral body axis of the Drosophila embryo. Development 124: 2741-2750.

20. Wappner P, Gabay L, Shilo BZ (1997) Interactions between the EGF receptor and DPP pathways establish distinct cell fates in the tracheal placodes. Development 124: 4707-4716.

21. Affolter M, Nellen D, Nussbaumer U, Basler K (1994) Multiple requirements for the receptor serine/threonine kinase thick veins reveal novel functions of TGF beta homologs during Drosophila embryogenesis. Development 120: 3105-3117.

22. Chihara T, Hayashi S (2000) Control of tracheal tubulogenesis by Wingless signaling. Development 127: 4433-4442.

23. Llimargas M (2000) Wingless and its signalling pathway have common and separable functions during tracheal development. Development 127: 4407-4417.

24. Jeon M, Zinn K (2009) Receptor tyrosine phosphatases control tracheal tube geometries through negative regulation of Egfr signaling. Development 136: 3121-3129.

25. Glazer L, Shilo BZ (2001) Hedgehog signaling patterns the tracheal branches. Development 128: 1599-1606.

26. Llimargas M (1999) The Notch pathway helps to pattern the tips of the Drosophila tracheal branches by selecting cell fates. Development 126: 2355-2364.

27. Ikeya T, Hayashi S (1999) Interplay of Notch and FGF signaling restricts cell fate and MAPK activation in the Drosophila trachea. Development 126: 4455-4463.

28. Englund C, Steneberg P, Falileeva L, Xylourgidis N, Samakovlis C (2002) Attractive and repulsive functions of Slit are mediated by different receptors in the Drosophila trachea. Development 129: 4941-4951.

29. Gallio M, Englund C, Kylsten P, Samakovlis C (2004) Rhomboid 3 orchestrates Slit-independent repulsion of tracheal branches at the CNS midline. Development 131: 3605-3614.

30. Lundstrom A, Gallio M, Englund C, Steneberg P, Hemphala J, et al. (2004) Vilse, a conserved Rac/Cdc42 GAP mediating Robo repulsion in tracheal cells and axons. Genes Dev 18: 2161-2171.

31. Brown S, Hu N, Hombria JC (2001) Identification of the first invertebrate interleukin JAK/STAT receptor, the Drosophila gene domeless. Curr Biol 11: 1700-1705.

32. Chen HW, Chen X, Oh SW, Marinissen MJ, Gutkind JS, et al. (2002) mom identifies a receptor for the Drosophila JAK/STAT signal transduction pathway and encodes a protein distantly related to the mammalian cytokine receptor family. Genes Dev 16: 388-398.

33. Chanut-Delalande H, Jung AC, Lin L, Baer MM, Bilstein A, et al. (2007) A genetic mosaic analysis with a repressible cell marker screen to identify genes involved in tracheal cell migration during Drosophila air sac morphogenesis. Genetics 176: 2177-2187.

34. Dammai V, Adryan B, Lavenburg KR, Hsu T (2003) Drosophila awd, the homolog of human nm23, regulates FGF receptor levels and functions synergistically with shi/dynamin during tracheal development. Genes Dev 17: 2812-2824.

35. Kakihara K, Shinmyozu K, Kato K, Wada H, Hayashi S (2008) Conversion of plasma membrane topology during epithelial tube connection requires Arf-like 3 small GTPase in Drosophila. Mech Dev 125: 325-336.

36. Chihara T, Kato K, Taniguchi M, Ng J, Hayashi S (2003) Rac promotes epithelial cell rearrangement during tracheal tubulogenesis in Drosophila. Development 130: 1419-1428.

37. Lee S, Kolodziej PA (2002) The plakin Short Stop and the RhoA GTPase are required for E-cadherin-dependent apical surface remodeling during tracheal tube fusion. Development 129: 1509-1520.

38. Jayaram SA, Senti KA, Tiklova K, Tsarouhas V, Hemphala J, et al. (2008) COPI vesicle transport is a common requirement for tube expansion in Drosophila. PLoS One 3: e1964.

39. Shindo M, Wada H, Kaido M, Tateno M, Aigaki T, et al. (2008) Dual function of Src in the maintenance of adherens junctions during tracheal epithelial morphogenesis. Development 135: 1355-1364.

40. Eulenberg KG, Schuh R (1997) The tracheae defective gene encodes a bZIP protein that controls tracheal cell movement during Drosophila embryogenesis. EMBO J 16: 7156-7165.

41. Ng T, Yu F, Roy S (2006) A homologue of the vertebrate SET domain and zinc finger protein Blimp-1 regulates terminal differentiation of the tracheal system in the Drosophila embryo. Dev Genes Evol 216: 243-252.

42. Guillemin K, Groppe J, Ducker K, Treisman R, Hafen E, et al. (1996) The pruned gene encodes the Drosophila serum response factor and regulates cytoplasmic outgrowth during terminal branching of the tracheal system. Development 122: 1353-1362.

43. Astle J, Kozlova T, Thummel CS (2003) Essential roles for the Dhr78 orphan nuclear receptor during molting of the Drosophila tracheal system. Insect Biochem Mol Biol 33: 1201-1209.

44. Jiang L, Crews ST (2006) Dysfusion transcriptional control of Drosophila tracheal migration, adhesion, and fusion. Mol Cell Biol 26: 6547-6556.

45. Dorfman R, Glazer L, Weihe U, Wernet MF, Shilo BZ (2002) Elbow and Noc define a family of zinc finger proteins controlling morphogenesis of specific tracheal branches. Development 129: 3585-3596.

46. Cubas P, Modolell J, Ruiz-Gomez M (1994) The helix-loop-helix extramacrochaetae protein is required for proper specification of many cell types in the Drosophila embryo. Development 120: 2555-2566.

47. Samakovlis C, Manning G, Steneberg P, Hacohen N, Cantera R, et al. (1996) Genetic control of epithelial tube fusion during Drosophila tracheal development. Development 122: 3531-3536.

48. Tanaka-Matakatsu M, Uemura T, Oda H, Takeichi M, Hayashi S (1996) Cadherin-mediated cell adhesion and cell motility in Drosophila trachea regulated by the transcription factor Escargot. Development 122: 3697-3705.

49. Hemphala J, Uv A, Cantera R, Bray S, Samakovlis C (2003) Grainy head controls apical membrane growth and tube elongation in response to Branchless/FGF signalling. Development 130: 249-258.

50. Myat MM, Lightfoot H, Wang P, Andrew DJ (2005) A molecular link between FGF and Dpp signaling in branch-specific migration of the Drosophila trachea. Dev Biol 281: 38-52.

51. Wolf C, Schuh R (2000) Single mesodermal cells guide outgrowth of ectodermal tubular structures in Drosophila. Genes Dev 14: 2140-2145.

52. Sedaghat Y, Miranda WF, Sonnenfeld MJ (2002) The jing Zn-finger transcription factor is a mediator of cellular differentiation in the Drosophila CNS midline and trachea. Development 129: 2591-2606.

53. Chen CK, Kuhnlein RP, Eulenberg KG, Vincent S, Affolter M, et al. (1998) The transcription factors KNIRPS and KNIRPS RELATED control cell migration and branch morphogenesis during Drosophila tracheal development. Development 125: 4959-4968.

54. Liu QX, Jindra M, Ueda H, Hiromi Y, Hirose S (2003) Drosophila MBF1 is a co-activator for Tracheae Defective and contributes to the formation of tracheal and nervous systems. Development 130: 719-728.

55. Han Z, Li X, Wu J, Olson EN (2004) A myocardin-related transcription factor regulates activity of serum response factor in Drosophila. Proc Natl Acad Sci U S A 101: 12567-12572.

56. Somogyi K, Rorth P (2004) Evidence for tension-based regulation of Drosophila MAL and SRF during invasive cell migration. Dev Cell 7: 85-93.

57. Wilk R, Reed BH, Tepass U, Lipshitz HD (2000) The hindsight gene is required for epithelial maintenance and differentiation of the tracheal system in Drosophila. Dev Biol 219: 183-196.

58. Bradley PL, Andrew DJ (2001) ribbon encodes a novel BTB/POZ protein required for directed cell migration in Drosophila melanogaster. Development 128: 3001-3015.

59. Shim K, Blake KJ, Jack J, Krasnow MA (2001) The Drosophila ribbon gene encodes a nuclear BTB domain protein that promotes epithelial migration and morphogenesis. Development 128: 4923-4933.

60. Centanin L, Dekanty A, Romero N, Irisarri M, Gorr TA, et al. (2008) Cell autonomy of HIF effects in Drosophila: tracheal cells sense hypoxia and induce terminal branch sprouting. Dev Cell 14: 547-558.

61. Lavista-Llanos S, Centanin L, Irisarri M, Russo DM, Gleadle JM, et al. (2002) Control of the hypoxic response in Drosophila melanogaster by the basic helix-loop-helix PAS protein similar. Mol Cell Biol 22: 6842-6853.

62. Kuhnlein RP, Schuh R (1996) Dual function of the region-specific homeotic gene spalt during Drosophila tracheal system development. Development 122: 2215-2223.

63. Franch-Marro X, Casanova J (2002) spalt-induced specification of distinct dorsal and ventral domains is required for Drosophila tracheal patterning. Dev Biol 250: 374-382.

64. Dorfman R, Shilo BZ, Volk T (2002) Stripe provides cues synergizing with branchless to direct tracheal cell migration. Dev Biol 252: 119-126.

65. Sonnenfeld M, Ward M, Nystrom G, Mosher J, Stahl S, et al. (1997) The Drosophila tango gene encodes a bHLH-PAS protein that is orthologous to mammalian Arnt and controls CNS midline and tracheal development. Development 124: 4571-4582.

66. Ward MP, Mosher JT, Crews ST (1998) Regulation of bHLH-PAS protein subcellular localization during Drosophila embryogenesis. Development 125: 1599-1608.

67. Wilk R, Weizman I, Shilo BZ (1996) trachealess encodes a bHLH-PAS protein that is an inducer of tracheal cell fates in Drosophila. Genes Dev 10: 93-102.

68. Isaac DD, Andrew DJ (1996) Tubulogenesis in Drosophila: a requirement for the trachealess gene product. Genes Dev 10: 103-117.

69. Araujo SJ, Cela C, Llimargas M (2007) Tramtrack regulates different morphogenetic events during Drosophila tracheal development. Development 134: 3665-3676.

70. Chiang C, Young KE, Beachy PA (1995) Control of Drosophila tracheal branching by the novel homeodomain gene unplugged, a regulatory target for genes of the bithorax complex. Development 121: 3901-3912.

71. Anderson MG, Certel SJ, Certel K, Lee T, Montell DJ, et al. (1996) Function of the Drosophila POU domain transcription factor drifter as an upstream regulator of breathless receptor tyrosine kinase expression in developing trachea. Development 122: 4169-4178.

72. Anderson MG, Perkins GL, Chittick P, Shrigley RJ, Johnson WA (1995) drifter, a Drosophila POU-domain transcription factor, is required for correct differentiation and migration of tracheal cells and midline glia. Genes Dev 9: 123-137.

73. Certel K, Anderson MG, Shrigley RJ, Johnson WA (1996) Distinct variant DNA-binding sites determine cell-specific autoregulated expression of the Drosophila POU domain transcription factor drifter in midline glia or trachea. Mol Cell Biol 16: 1813-1823.

74. Zelzer E, Shilo BZ (2000) Interaction between the bHLH-PAS protein Trachealess and the POU-domain protein Drifter, specifies tracheal cell fates. Mech Dev 91: 163-173.

75. Englund C, Uv AE, Cantera R, Mathies LD, Krasnow MA, et al. (1999) adrift, a novel bnl-induced Drosophila gene, required for tracheal pathfinding into the CNS. Development 126: 1505-1514.

76. Paul SM, Ternet M, Salvaterra PM, Beitel GJ (2003) The Na+/K+ ATPase is required for septate junction function and epithelial tube-size control in the Drosophila tracheal system. Development 130: 4963-4974.

77. Mortimer NT, Moberg KH (2007) The Drosophila F-box protein Archipelago controls levels of the Trachealess transcription factor in the embryonic tracheal system. Dev Biol 312: 560-571.

78. Krause C, Wolf C, Hemphala J, Samakovlis C, Schuh R (2006) Distinct functions of the leucine-rich repeat transmembrane proteins capricious and tartan in the Drosophila tracheal morphogenesis. Dev Biol 296: 253-264.

79. Hartenstein K, Sinha P, Mishra A, Schenkel H, Torok I, et al. (1997) The congested-like tracheae gene of Drosophila melanogaster encodes a member of the mitochondrial carrier family required for gas-filling of the tracheal system and expansion of the wings after eclosion. Genetics 147: 1755-1768.

80. Matusek T, Djiane A, Jankovics F, Brunner D, Mlodzik M, et al. (2006) The Drosophila formin DAAM regulates the tracheal cuticle pattern through organizing the actin cytoskeleton. Development 133: 957-966.

81. Jazwinska A, Ribeiro C, Affolter M (2003) Epithelial tube morphogenesis during Drosophila tracheal development requires Piopio, a luminal ZP protein. Nat Cell Biol 5: 895-901.

82. Mortimer NT, Moberg KH (2009) Regulation of Drosophila embryonic tracheogenesis by dVHL and hypoxia. Dev Biol 329: 294-305.

83. Adryan B, Decker HJ, Papas TS, Hsu T (2000) Tracheal development and the von Hippel-Lindau tumor suppressor homolog in Drosophila. Oncogene 19: 2803-2811.

84. Van Doren M, Mathews WR, Samuels M, Moore LA, Broihier HT, et al. (2003) fear of intimacy encodes a novel transmembrane protein required for gonad morphogenesis in Drosophila. Development 130: 2355-2364.

85. Steneberg P, Samakovlis C (2001) A novel stop codon readthrough mechanism produces functional Headcase protein in Drosophila trachea. EMBO Rep 2: 593-597.

86. Sarkar S, Lakhotia SC (2005) The Hsp60C gene in the 25F cytogenetic region in Drosophila melanogaster is essential for tracheal development and fertility. J Genet 84: 265-281.

87. Devine WP, Lubarsky B, Shaw K, Luschnig S, Messina L, et al. (2005) Requirement for chitin biosynthesis in epithelial tube morphogenesis. Proc Natl Acad Sci U S A 102: 17014-17019.

88. Moussian B, Tang E, Tonning A, Helms S, Schwarz H, et al. (2006) Drosophila Knickkopf and Retroactive are needed for epithelial tube growth and cuticle differentiation through their specific requirement for chitin filament organization. Development 133: 163-171.

89. Tonning A, Hemphala J, Tang E, Nannmark U, Samakovlis C, et al. (2005) A transient luminal chitinous matrix is required to model epithelial tube diameter in the Drosophila trachea. Dev Cell 9: 423-430.

90. Llimargas M, Strigini M, Katidou M, Karagogeos D, Casanova J (2004) Lachesin is a component of a septate junction-based mechanism that controls tube size and epithelial integrity in the Drosophila tracheal system. Development 131: 181-190.

91. Guillemin K, Williams T, Krasnow MA (2001) A nuclear lamin is required for cytoplasmic organization and egg polarity in Drosophila. Nat Cell Biol 3: 848-851.

92. Behr M, Riedel D, Schuh R (2003) The claudin-like megatrachea is essential in septate junctions for the epithelial barrier function in Drosophila. Dev Cell 5: 611-620.

93. Page-McCaw A, Serano J, Sante JM, Rubin GM (2003) Drosophila matrix metalloproteinases are required for tissue remodeling, but not embryonic development. Dev Cell 4: 95-106.

94. Araujo SJ, Aslam H, Tear G, Casanova J (2005) mummy/cystic encodes an enzyme required for chitin and glycan synthesis, involved in trachea, embryonic cuticle and CNS development--analysis of its role in Drosophila tracheal morphogenesis. Dev Biol 288: 179-193.

95. Beitel GJ, Krasnow MA (2000) Genetic control of epithelial tube size in the Drosophila tracheal system. Development 127: 3271-3282.

96. Zhang S, Dailey GM, Kwan E, Glasheen BM, Sroga GE, et al. (2006) An MMP liberates the Ninjurin A ectodomain to signal a loss of cell adhesion. Genes Dev 20: 1899-1910.

97. Liu L, Johnson WA, Welsh MJ (2003) Drosophila DEG/ENaC pickpocket genes are expressed in the tracheal system, where they may be involved in liquid clearance. Proc Natl Acad Sci U S A 100: 2128-2133.

98. Inagaki S, Numata K, Kondo T, Tomita M, Yasuda K, et al. (2005) Identification and expression analysis of putative mRNA-like non-coding RNA in Drosophila. Genes Cells 10: 1163-1173.

99. Zhang SD, Kassis J, Olde B, Mellerick DM, Odenwald WF (1996) Pollux, a novel Drosophila adhesion molecule, belongs to a family of proteins expressed in plants, yeast, nematodes, and man. Genes Dev 10: 1108-1119.

100. Jung AC, Ribeiro C, Michaut L, Certa U, Affolter M (2006) Polychaetoid/ZO-1 is required for cell specification and rearrangement during Drosophila tracheal morphogenesis. Curr Biol 16: 1224-1231.

101. Moussian B, Soding J, Schwarz H, Nusslein-Volhard C (2005) Retroactive, a membrane-anchored extracellular protein related to vertebrate snake neurotoxin-like proteins, is required for cuticle organization in the larva of Drosophila melanogaster. Dev Dyn 233: 1056-1063.

102. Luschnig S, Batz T, Armbruster K, Krasnow MA (2006) serpentine and vermiform encode matrix proteins with chitin binding and deacetylation domains that limit tracheal tube length in Drosophila. Curr Biol 16: 186-194.

103. Wang S, Jayaram SA, Hemphala J, Senti KA, Tsarouhas V, et al. (2006) Septate-junction-dependent luminal deposition of chitin deacetylases restricts tube elongation in the Drosophila trachea. Curr Biol 16: 180-185.

104. Wu VM, Yu MH, Paik R, Banerjee S, Liang Z, et al. (2007) Drosophila Varicose, a member of a new subgroup of basolateral MAGUKs, is required for septate junctions and tracheal morphogenesis. Development 134: 999-1009.

105. Behr M, Wingen C, Wolf C, Schuh R, Hoch M (2007) Wurst is essential for airway clearance and respiratory-tube size control. Nat Cell Biol 9: 847-853.
