# Supplementary material for: A Systematic Screen for Tube Morphogenesis and Branching Genes in the Drosophila Tracheal System
Source: PLoS Genet. 2011 Jul 7;7(7):e1002087. doi: 10.1371/journal.pgen.1002087 (PMC3131284; doi:10.1371/journal.pgen.1002087)
Supplement: Table S2 — Polymerase chain reaction (PCR) primers. Primers were used to generate the modified pUAST vector, pUASTi, and the GFP(RNAi) construct, pUASTi-GFPhp. (DOC) [file pgen.1002087.s003.doc]

# Table S2. Polymerase chain reaction (PCR) primers

*Primers for trachealess intron*

Xho+trh-intron F – 5'CTCGAGCAGGTAAGATATGGTAGCG

Kpn+trh-intron B – 5'GGTACCCCAGCGACTGCAGAAAATG

*Primers for GFP RNAi construct*

Not-GFP-F: GCGGCCGCTGGAGAGGGTGAAGGTGATGC

Xho-GFP-R: CTCGAGAGATTGTGTGGACAGGTAATGGTTG

Xba-GFP-F: TCTAGATGGAGAGGGTGAAGGTGATGC

Kpn-GFP-R: GGTACCAGATTGTGTGGACAGGTAATGGTTG
