# Supplementary material for: A Systematic Screen for Tube Morphogenesis and Branching Genes in the Drosophila Tracheal System
Source: PLoS Genet. 2011 Jul 7;7(7):e1002087. doi: 10.1371/journal.pgen.1002087 (PMC3131284; doi:10.1371/journal.pgen.1002087)
Supplement: Table S3 — Additional tracheal morphogenesis mutants. Genes that were identified in the screen but represented by a single allele, and for which the molecular identity of the gene remains unknown. Abbreviations and gene mapping methods are given in the footnotes of Table 1. The estimate of 70 tracheal genes identified in our screen includes the 58 named loci in Table 1 plus the 12 loci in this table (PC146, 137, 198, 826, 889, 928, 1055, 1106, 1631, 1663, 1801) in which a mapped lethal mutation was identified by deficiency mapping (see “Map Position”). However, these mapped lethal mutations may not be in all cases the mutation responsible for the tracheal phenotype. (DOC) [file pgen.1002087.s004.doc]

# Table S3. Additional tracheal morphogenesis mutants

| **Name (alleles)** | **Tracheal Phenotype (category) [other affects]** | **Map Position**  **(method)** |
| --- | --- | --- |
| (AA36) | DT breaks | Ch 3 |
| (AB95) | Short DT | Ch 3 |
| (AE18) | DT breaks | Ch 3 |
| (AI14) | DT breaks | Ch 3 |
| (AQ7) | Pruned TC (3A) without gas-filling | Ch 3 |
| (AY29) | DT lumen collapse | Ch 3 |
| (BB81) | Mild to moderate TC pruning (3A) | 3L  (MA) |
| (BG5) | DT breaks | Ch 3 |
| (BI9) | Low GFP reporter expression | Ch 3 |
| (BO78) | Low GFP reporter expression | Ch 3 |
| (PC146) | Mild TC pruning (3A) and variable gas-filling defect. Autocellular tubes with variable gas-filling defect. | 87B-D & 100A; C  (Df 34,35,48) |
| (137) | Mild TC pruning (3A) | 89E; 90C & 94E  (Df 49,50) |
| (198) | Mild to severe TC pruning (3A) | 64C; 65C  (Df 42) |
| (635) | Mild to moderate TC pruning (3A) with strong gas-filling defect | 3R  (MA) |
| (777) | TC with variable gas-filling defect (5A1) | 3R  (MA) |
| (826) | TC with gas-filling defect at branch tips (5A2) and possible mild pruning. [Rough eyes, inflated hairy wings *in trans* to Df 10] | 89E11; 90A7  (Df 46,49) |
| (889) | Weak TC pruning defect (5A1) and variable gas-filling | 63E; 64A  (Df 54) |
| (924) | TC show moderate to strong pruning defect (3A) | 3L  (MA) |
| (928) | Weak and variable TC branch tip gas-filling defect (5A2). [Wings held out *in trans* to Df 57] | 95F; 96A  (Df 9) |
| (1030) | Moderate to severe TC pruning (3C). Resembles but complements *denuded.* | 3R  (MA) |
| (1055) | Mild TC pruning (3A) | 69A2; 69D1  (Df 43) |
| (1106**)** | TC gas-filling defect (5A1) | 83E; 84A  (Df 53) |
| (1163) | Severe TC pruning (3A) | 3L  (MA) |
| (1301) | Mild to moderate TC pruning (3A) with variable gas-filling defect and large vacuoles. [Reduced, rough eyes *in trans* to Df 58] | 87D; 87E, 95F; 96A, 97A; 98A, &  100A; 100C  (Df 9,35,38,48,54) |
| (1407) | Mild to moderate TC pruning (3A) | 3L  (MA) |
| (1631) | TC gas-filling defect (5A1) | 99D; 99E  (Df 55) |
| (1637) | Moderate to severe TC pruning (3A) with gas-filling defect. LumGFP is not retained in mutant clones. DsRED is faint and punctate in mutant cells. | 3L  (MA) |
| (1663) | Moderate to severe TC pruning (3A) with gas-filling defect | 67A; 67D  (Df 56) |
| (1667) | Moderate to severe TC pruning (3A) with gas-filling defect. LumGFP is not retained in mutant clones. DsRED is faint and punctate in mutant cells. | 3L  (MA) |
| (1680) | Mild to moderate TC pruning (3A) with variable gas-filling defect. [Wing vein deltas *in trans* to Df 2] | 3R  (MA) |
| (1697) | Mild to moderate TC pruning (3A) with narrow bore seamless tubes with gas-filling defect | 3R  (MA) |
| (1801) | Moderate TC pruning (3A) | 83E; 84A  (Df 53) |
| (1809) | Weak TC gas-filling defect (5A2) | 3L  (MA) |
| (1837) | Mild to moderate TC pruning (3A) with variable gas-filling defect. GFP reporter levels elevated in clone. | 3L  (MA) |
